# Unisexual Reproduction Drives Meiotic Recombination and Phenotypic and Karyotypic Plasticity in *Cryptococcus neoformans*

**DOI:** 10.1371/journal.pgen.1004849

**Published:** 2014-12-11

**Authors:** Sheng Sun, R. Blake Billmyre, Piotr A. Mieczkowski, Joseph Heitman

**Affiliations:** 1 Department of Molecular Genetics and Microbiology, Duke University Medical Center, Durham, North Carolina, United States of America; 2 Department of Biology, High-Throughput Sequencing Facility, University of North Carolina at Chapel Hill, Chapel Hill, North Carolina, United States of America; University College Dublin, Ireland

## Abstract

In fungi, unisexual reproduction, where sexual development is initiated without the presence of two compatible mating type alleles, has been observed in several species that can also undergo traditional bisexual reproduction, including the important human fungal pathogens *Cryptococcus neoformans* and *Candida albicans*. While unisexual reproduction has been well characterized qualitatively, detailed quantifications are still lacking for aspects of this process, such as the frequency of recombination during unisexual reproduction, and how this compares with bisexual reproduction. Here, we analyzed meiotic recombination during α-α unisexual and **a**-α bisexual reproduction of *C. neoformans*. We found that meiotic recombination operates in a similar fashion during both modes of sexual reproduction. Specifically, we observed that in α-α unisexual reproduction, the numbers of crossovers along the chromosomes during meiosis, recombination frequencies at specific chromosomal regions, as well as meiotic recombination hot and cold spots, are all similar to those observed during **a**-α bisexual reproduction. The similarity in meiosis is also reflected by the fact that phenotypic segregation among progeny collected from the two modes of sexual reproduction is also similar, with transgressive segregation being observed in both. Additionally, we found diploid meiotic progeny were also produced at similar frequencies in the two modes of sexual reproduction, and transient chromosomal loss and duplication likely occurs frequently and results in aneuploidy and loss of heterozygosity that can span entire chromosomes. Furthermore, in both α-α unisexual and **a**-α bisexual reproduction, we observed biased allele inheritance in regions on chromosome 4, suggesting the presence of fragile chromosomal regions that might be vulnerable to mitotic recombination. Interestingly, we also observed a crossover event that occurred within the *MAT* locus during α-α unisexual reproduction. Our results provide definitive evidence that α-α unisexual reproduction is a meiotic process similar to **a**-α bisexual reproduction.

## Author Summary

Unisexual reproduction has been reported in several fungal species that have been traditionally thought to undergo bisexual reproduction, including major human fungal pathogens such as *Cryptococcus neoformans* and *Candida albicans*. While it has been well characterized qualitatively, quantitative description of unisexual reproduction, and detailed comparisons between unisexual and bisexual reproduction, are lacking. Here, by analyzing meiotic progeny generated from both α-α unisexual and **a**-α bisexual reproduction in *C. neoformans*, we find that the progeny collected from the two modes of sexual reproduction show similar phenotypic segregation, with transgressive segregation of several phenotypes being observed in both. Additionally, the two modes of sexual reproduction are similar in all the aspects of meiotic recombination that we have examined, providing definitive evidence that α-α unisexual reproduction is a meiotic process that operates similarly as in **a**-α bisexual reproduction. The ability to undergo both unisexual and bisexual reproduction may provide evolutionary advantages in environments where suitable mating partners are scarce, or where sexual reproduction is favored over asexual reproduction by mixing genetic materials and producing spores that are more tolerant of harsh environments. We discuss the implications of these findings in the context of the evolution of pathogenesis, mating types, and mating systems.

## Introduction

*Cryptococcus neoformans* is a globally distributed basidiomycetous human fungal pathogen that causes life threatening meningoencephalitis [1]. *C. neoformans* predominantly infects individuals with compromised immunity, such as HIV/AIDS patients, and now causes more than one million cases of cryptococcosis and >620,000 attributable mortalities globally, and accounts for approximately one-third of all AIDS-associated deaths each year [2].

*C. neoformans* typically reproduces asexually in the environment as a haploid, budding yeast. When suitable conditions arise, it can also undergo sexual reproduction [3], [4], [5], [6], [7]. There are two mating types in *C. neoformans*, **a** and α, which are defined by the presence of two alternate alleles of the mating type locus (*MAT* **a** and *MAT*α) [8]. In response to a variety of environmental cues, **a** and α cells undergo a well characterized bisexual reproduction cycle in which cells of opposite mating type first undergo cell-cell fusion that is evoked by pheromone secretion and sensing. The resulting dikaryon transitions into hyphal growth and eventually the hyphal tips form basidia fruiting bodies in which nuclear fusion and one round of meiosis occur [9]. After meiosis, multiple rounds of mitosis and budding produce on the surface of each basidium four long chains of infectious spores, which can be readily aerosolized and cause infections in mice and possibly also humans [10].

Although the natural population of *C. neoformans* is clonal overall, signatures of recombination have been detected, suggesting mating indeed occurs in nature, albeit at a low frequency or involving inbreeding [1]. In laboratory crosses, it has been found that during *C. neoformans* sexual reproduction, meiotic recombination is not completely random, and recombination hot spots and cold spots have been identified [11], [12], [13]. The *MAT* locus of *C. neoformans* is unusually large (>100 kb) and the two mating type alleles, *MAT* **a** and *MAT*α, have similar gene contents. However, extensive chromosomal rearrangements have accumulated between the *MAT* **a** and *MAT*α alleles, and consequently, meiotic recombination is generally highly repressed within the *MAT* locus during sexual reproduction, presumably due to sequence divergence and chromosomal rearrangements that would lead to dicentric/acentric chromosomes if crossing over were to occur. Interestingly, while recombination is suppressed within the *MAT* locus, two GC-rich recombination hot spots have been identified that flank the *MAT* locus [11]. Additionally, a minor GC-rich intergenic region located within the *MAT* locus stimulates recombination via gene conversion rather than crossing over [12].

The *C. neoformans* natural population has an extremely biased mating type distribution, and α mating type accounts for >99% of the natural isolates analyzed [14]. This is very striking, and also posed a conundrum: How frequently does sexual reproduction between **a** and α cells occur in nature, given a population with such an unbalanced mating type ratio? Additionally, how important is sexual reproduction in the evolution of *C. neoformans*, if it is not occurring at a high enough frequency, especially considering that sex also comes with intrinsic costs compared to asexual reproduction [15]?

Answers to these questions were provided by a previous study which found that *C. neoformans* can undergo not only **a**-α bisexual reproduction, but also α-α unisexual reproduction involving cells of only one mating type [16]. The developmental aspects of α-α unisexual reproduction mirror those of **a**-α bisexual reproduction. Although a detailed description of recombination frequencies during α-α unisexual reproduction is as yet lacking, it has been shown that genes essential for meiosis and sporulation are also required for successful α-α unisexual reproduction, providing evidence that unisexual reproduction in *C. neoformans* involves meiosis [16], [17]. Additionally, natural αAAα and αADα *C. neoformans* isolates that are diploid and have two *MAT*α alleles have been isolated from the environment and clinic, providing further evidence that α-α unisexual reproduction occurs in natural populations [18], [19]. Furthermore, α-α unisexual reproduction has also been suggested to occur in nature based on several population genetics studies of *C. neoformans* [20], [21], [22], [23]. Moreover, natural αADα isolates show hybrid vigor and exhibit greater fitness compared to haploid serotype A and serotype D strains [18]. Together, these studies support the model that α-α unisexual reproduction is ongoing in natural *C. neoformans* populations, and could be beneficial and evolutionarily advantageous.

Interestingly, unisexual reproduction has been recently discovered to occur in *C. albicans*, another common human fungal pathogen [24]. *C. albicans* is known to undergo a parasexual cycle involving fusion between cells of opposite mating types, **a** and α, followed by stochastic, concerted loss of chromosomes that eventually restores the diploid state [25]. The study by Alby et al. [24] found that when Bar1, a pheromone-degrading protease, is inactivated, a homothallic parasexual cycle can also be initiated in *C. albicans* via **a**-**a** cell fusion.

The discovery of an expanded ability to undergo both unisexual and bisexual reproduction in two leading fungal pathogens indicates that the benefits of sex may outweigh the costs in these species. This is also supported by recent studies in which it has been shown that in *C. neoformans*, sexual reproduction, including both unisexual and bisexual, frequently generates aneuploid progeny that have altered fitness under a variety of conditions [26].

Although α-α unisexual reproduction in *C. neoformans* has been well characterized qualitatively, several fundamental aspects of this unique process are yet to be described quantitatively. The objective of our study was to conduct a detailed comparison between α-α unisexual and **a**-α bisexual reproduction. Specific questions we addressed are: Does meiotic recombination indeed occur during α-α unisexual reproduction? If so, what are the recombination frequencies during α-α unisexual reproduction, and how does this compare to **a**-α bisexual reproduction? Also, how does the recombination frequency profile in α-α unisexual reproduction compare to that observed in **a**-α bisexual reproduction? Do recombination hot spots also operate during α-α unisexual reproduction? Additionally, does recombination, especially crossing over, occur within the *MAT* locus during α-α unisexual reproduction, given that now the physical restraints imposed by chromosomal divergence/rearrangements during **a**-α bisexual reproduction are relaxed? Furthermore, are the products of α-α unisexual reproduction strictly haploid F1 meiotic recombinants, or are aneuploids and diploids also produced as products and not only intermediates in the pathway? Answers to these questions will provide a better understanding of the evolution of the *MAT* locus and sexual reproduction, as well as the importance of sexual reproduction in shaping population dynamics and evolutionary trajectories of not only *C. neoformans*, but also other fungal species and beyond.

## Results

### Mating products were recovered from both α-α unisexual and a-α bisexual reproduction

One of the main objectives of our study was to investigate the frequency of recombination during α-α unisexual reproduction in *C. neoformans*, and how this compares to **a**-α bisexual reproduction. To achieve this, we first set up and recovered mating products from both unisexual and bisexual reproduction.

To study meiotic recombination during α-α unisexual reproduction, we crossed two *MAT*α strains, 431α (*URA5 NAT^S^*) and XL280αSS (*ura5 NAT^R^*), and then screened for recombinant mating products that are *URA5* and *NAT^R^*. We isolated mating products from α-α unisexual reproduction by screening for recombinant progeny using phenotypic markers instead of by spore dissection for the following reasons. First, as opposed to **a**-α bisexual reproduction, α-α meiotic recombination has not been quantified previously. Thus, we wanted to obtain a large progeny population that had undergone as much recombination as possible, which would make our estimation of recombination frequency as robust as possible. This was achieved efficiently by screening for recombinants using phenotypic markers. Second, it has been shown that the strain XL280α can undergo selfing and produce spores, albeit at lower frequency compared to typical **a**-α bisexual reproduction. Thus, by screening for recombinants using phenotypic markers we could avoid including progeny originating from XL280α selfing, which would otherwise distort the estimation of recombination frequency of α-α unisexual reproduction. Additionally, and importantly, the markers that we used, *URA5* and NAT, are located on different chromosomes, and neither is located on chromosome 4. As such, this screening process would not affect our analysis of meiotic recombination frequency during α-α unisexual reproduction. In total, from α-α unisexual reproduction we recovered a total of 156 progeny that were *URA5* NAT^R^, representing mating products of the two parental strains.

For comparison, using the same strain 431α as one of the two parents, we also conducted an **a**-α bisexual reproduction by crossing it with strain XL280**a**, which is congenic with the XL280α parental strain used for unisexual reproduction [27], except for the *MAT* locus and the loci where the *ura5* and NAT^R^ markers are located. This is also supported by CHEF gel electrophoresis in which no apparent karyotypic variation has been observed between strains XL280**a** and XL280αSS (S4 Figure). Thus, by crossing the same natural isolate with two isogenic **a** and α strains, we could eliminate possible complications due to karyotypic and sequence differences between parental strains used in the two types of crosses, and conduct a more consistent evaluation and comparison of recombination frequencies between the two modes of sexual reproduction. Compared to α-α unisexual reproduction, basidia and basidiospore chains were produced more abundantly during **a**-α bisexual reproduction. In this case, meiotic products from **a**-α bisexual reproduction were collected by dissecting basidiospores from an individual basidium. Spores from twenty-nine basidia were dissected from **a**-α bisexual reproduction, with varying spore germination rates among basidia that ranged between 0% (basidia 8 and 23) and 100% (basidium 2) (Table 1). In total, 261 basidiospores were recovered from 27 basidia for **a**-α bisexual reproduction.

**Table 1:** Summary of basidia dissected from opposite sex mating between strains 431α and XL280a.

Basidium No.	No. of spores dissected	No. of spores germinated	Germination rate (%)	Progeny^1^
1	17	7	41	SSB859–SSB865
2	9	9	100	SSB866–SSB874
3	21	11	52	SSB875–SSB885
4	20	3	15	SSB886–SSB888
5	39	24	62	SSB889–SSB912
6	33	28	85	SSB913–SSB940
7	32	17	53	SSB941–SSB957
8	22	0	0	n.a.^3^
9	14	5	36	SSB958–SSB962
10 & 11^2^	44	12	27	SSB963–SSB974
12	12	10	83	SSB975–SSB984
13 & 14^2^	17	16	94	SSB985–SSB1000
15	4	1	25	SSC001
16	26	24	92	SSC002–SSC025
17	11	4	36	SSC026–SSC029
18	9	2	22	SSC030–SSC031
19	20	13	65	SSC032–SSC044
20	13	6	46	SSC045–SSC050
21	14	13	93	SSC228–SSC240
22	10	2	20	SSC241–SSC242
23	12	0	0	n.a.^3^
24 & 25^2^	39	28	72	SSC243–SSC270
26	17	2	12	SSC271–SSC272
27	20	5	25	SSC273–SSC277
28	14	12	86	SSC278–SSC289
29	28	7	25	SSC290–SSC296

1 : The strain names correspond to those in S4 Table.

2 : Mixture of basidiospores from two basidia.

3 : No spore germinated.

### Phenotypic variation is observed in progeny from both α-α unisexual and a-α bisexual reproduction

We first investigated phenotypic variation among the progeny generated through α-α unisexual and **a**-α bisexual reproduction. The phenotypes that we assessed were temperature tolerance on YPD solid medium at different temperatures, melanin production on L-DOPA solid medium, and hyphal production on MS solid medium.

**Figure 1:**
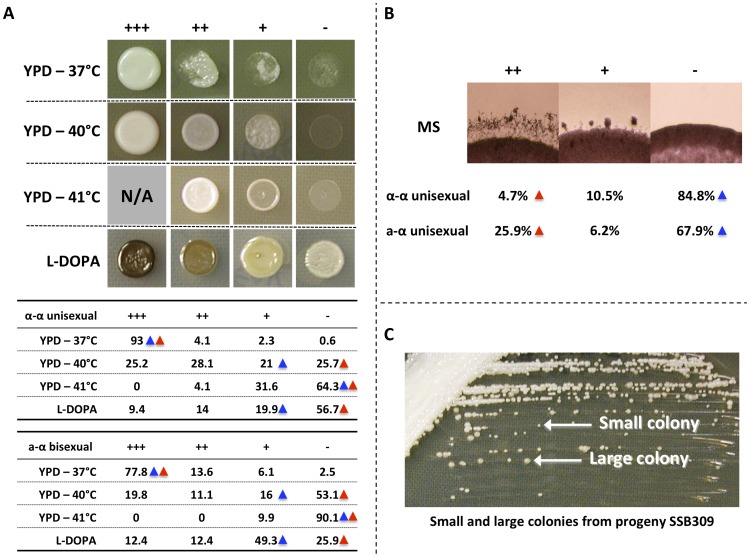
Phenotypic analyses of progeny from α-α unisexual and a-α bisexual reproduction. A) Top: Different phenotypic categories that were observed among the meiotic progeny from α-α unisexual and **a**-α bisexual reproduction. Bottom: Summary of percentage (%) of progeny that belonged to different phenotypic categories in α-α unisexual and **a**-α bisexual reproduction. Blue triangles highlight the phenotypes of parental strain 431α; red triangles highlight the phenotypes of the parental strains XL280αSS and XL280**a**. B) Phenotypic segregation of hyphal growth among the meiotic progeny from α-α unisexual and **a**-α bisexual reproduction. The numbers represent the percentage of progeny that belong to different phenotypic categories. Blue triangles highlight the phenotypes of parental strain 431α; red triangles highlight the phenotypes of the parental strains XL280αSS and XL280**a**. C) The small and large colonies of the meiotic progeny SSB309 when grown on YPD solid medium at 30°C.

For temperature tolerance, we tested the growth of the progeny on YPD medium at three temperatures: 37°C, 40°C, and 41°C (S1 Table and S2 Table). At each temperature we found progeny from both α-α unisexual and **a**-α bisexual reproduction that exhibited transgressive phenotypes compared to the two parental strains. Specifically, at 37°C none of the parental strains showed a growth defect. However, we found 7% and 22.2% of the progeny from α-α unisexual and **a**-α bisexual reproduction, respectively, exhibited growth defect, and thus are sensitive to high temperature (Fig. 1A). At 40°C and 41°C, the three parental strains showed little or no growth. However, there were 53.3% and 35.7% of the progeny from α-α unisexual reproduction that showed enhanced fitness at 40°C and 41°C, respectively. Similarly, from **a**-α unisexual reproduction, there were also 30.9% and 9.9% of the progeny that showed enhanced fitness at 40°C and 41°C, respectively (Fig. 1A).

For melanin production on L-DOPA medium, we also found progeny that exhibit transgressive phenotype compared to the parental strains (S1 Table and S2 Table). Specifically, we found that 23.4% progeny from α-α unisexual reproduction, and 24.8% progeny from **a**-α bisexual reproduction, showed elevated melanin production compared to their parental strains, respectively (Fig. 1A).

For hyphal production on MS medium, we did not observe transgressive segregation among progeny from either α-α unisexual or **a**-α bisexual reproduction (S1 Table and S2 Table). Instead, we found that 10.5% and 6.2% of the progeny showed intermediate hyphal production compared to their parental strains in α-α unisexual or **a**-α bisexual reproduction, respectively.

### Meiotic recombination occurs during α-α unisexual reproduction

**Figure 2:**
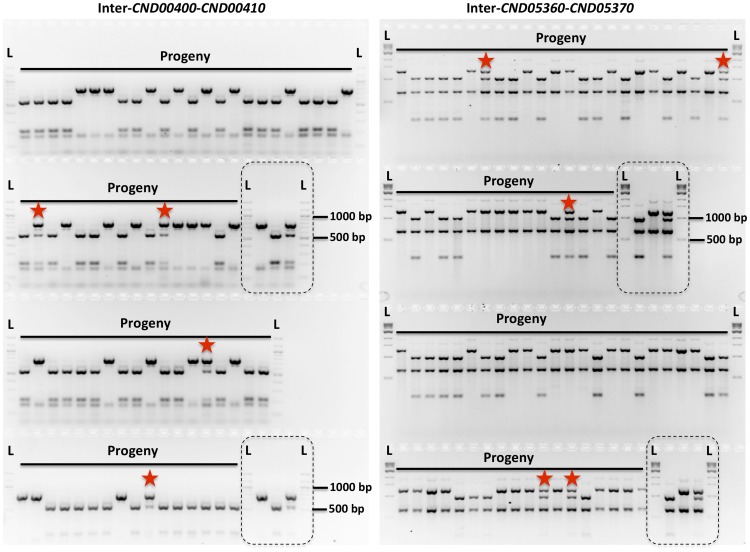
Genetic markers and genotyping of the meiotic progeny. Shown here are results of genotyping of meiotic progeny from α-α unisexual reproduction strains 431α and XL280αSS. The marker on the left is “Inter-*CND00400*–*CND00410*” and the marker on the right is “Inter-*CND05360*–*CND05370*” (Please see Table 2 for detailed marker information). Dashed-lined rectangles highlight the controls included during genotyping, which are (from left to right): 431α, XL280αSS, and artificial heterozygote (i.e. mixed DNA sample; please see Materials and Methods for detailed information). “L” indicates DNA ladder. The progeny that are heterozygous at these two markers are highlighted with red stars.

To study meiotic recombination during α-α unisexual and **a**-α bisexual reproduction, we focused on chromosome 4 where the *MAT* locus is located. We developed 44 co-dominant genetic markers that are located along chromosome 4, including eight markers that are located within the *MAT* locus, as well as two markers that flank the centromere of chromosome 4 (Table 2; Fig. 2).

**Table 2:** Genetic markers employed for genotyping progeny from α-α unisexual and a-α bisexual reproduction.

Marker Name^1^	Serial No. (α-α unisexual)	Serial No. (a-α bisexual)	Location^1^	Primer (Forward)	Primer (Reverse)	Enzyme	Note
Inter-*CND00070*–*CND00080*	1		20927	JOHE40225	JOHE40226	HaeIII	
Inter-*CND00310*–*CND00320*	2		96186	JOHE40241	JOHE40242	BstXI	
Inter-*CND00400*–*CND00410*	3		120603	JOHE40245	JOHE40246	BccI	
Inter-*CND00490*–*CND00500*	4	1	147672	JOHE40255	JOHE40256	HaeIII	
Inter-*CND00610*–*CND00620*	5		173409	JOHE40261	JOHE40262	BccI	
Chrom4_220 kb	6	2	220450	JOHE40191	JOHE40192	HphI	CEN flanking
Chrom4_280 kb	7	3	280450	JOHE40215	JOHE40216	Hpy188I	CEN flanking
Inter-*CND01130*–*CND01140*	8	4	331643	JOHE26611	JOHE26696	HhaI	
Inter-*CND01270*–*CND01280*	9	5	362686	JOHE26625	JOHE26710	HaeIII	
Inter-*CND01480*–*CND01490*	10	6	416408	JOHE40269	JOHE40270	NciI	
Inter-*CND01730*–*CND01740*	11		468458	JOHE26670	JOHE26755	AatII	
Inter-*CND02320*–*CND02330*	12		623225	JOHE40283	JOHE40284	AciI	
Inter-*CND02440*–*CND02450*	13		662365	JOHE40287	JOHE40288	HinfI	
*CND02580*	14	7	704101	JOHE23347	JOHE23348	NciI	
Inter-*CND02890*–*CND02900*	15		784987	JOHE40295	JOHE40296	BsrFI	
Inter-*CND03060*–*CND03070*	16		830051	JOHE40299	JOHE40300	HaeIII	
Inter-*CND03160*–*CND03170*	17		854912	JOHE40301	JOHE40302	BsaHI	
Inter-*CND03240*–*CND03250*	18		878777	JOHE40303	JOHE40304	AccI	
*CND03480*	19		944503	JOHE23353	JOHE23354	HphI	
*CND03960*	20		1096841	JOHE23357	JOHE23358	AflIII	
Inter-*CND04040*–*CND04050*	21	8	1129340	JOHE40317	JOHE40318	EcoRV	
*CND04340*	22		1203443	JOHE23361	JOHE23362	Sau96I	
*CND04760*	23		1307202	JOHE27442	JOHE27443	Sau96I	
Inter-*CND04980*–*CND04990*	24	9	1365745	JOHE27494	JOHE27495	EcoRV	
Inter-*CND05010*–*CND05020*	25		1372992	JOHE27500	JOHE27501	HinfI	
*CND05140*	26		1407510	JOHE23367	JOHE23368	TfiI	
*CND05310*	27		1447724	JOHE23369	JOHE23370	AccI	
Inter-*CND05360*–*CND05370*	28		1469659	JOHE40007	JOHE40008	NciI	
Inter-*CND05440*–*CND05450*	29	10	1480737	JOHE40021	JOHE40022	Sau96I	
Inter-*CND05590*–*CND05600*	30	11	1514191	JOHE40043	JOHE40044	EcoRV	
Inter-*BSP3*-*IKS1*	31	12	1529240	JOHE39948	JOHE39949	NciI	*MAT*
*ZNF1*	32		1546466	JOHE38697	JOHE38698	BstYI	*MAT*
Inter-*STE20*-*MYO2*	33		1571084	JOHE39932	JOHE39933	MseI	*MAT*
Inter-*LTRTcn10*-*LTRCnirt3*	34		1575936	JOHE39928	JOHE39929	Sau96I	*MAT*
Inter-*STE3*-*LTRTcn10*	35		1577047	JOHE39926	JOHE39927	SphI	*MAT*
Inter-*RPO41*-*STE12*	36		1584015	JOHE39922	JOHE39923	DdeI	*MAT*
Inter-*RUM1*-*GEF1*	37		1602717	JOHE39912	JOHE39913	MspI	*MAT*
*SXI1*Dα	38	13α^2^	1637942	JOHE17409	JOHE14895	n.a.^3^	*MAT*
*SXI2*D**a**	n.a.^4^	13**a** 2	n.a.^5^	JOHE39965	JOHE39966	n.a.^3^	*MAT*
Inter-*CND06020*–*CND06030*	39	14	1661861	JOHE40047	JOHE40048	DdeI	
Inter-*CND06040*–*CND06050*	n.a.^4^	15	1665521	JOHE40049	JOHE40050	AciI	
Inter-*CND06130*–*CND06140*	40	16	1693348	JOHE40067	JOHE40068	HphI	
Inter-*CND06290*–*CND06300*	41	17	1742645	JOHE40087	JOHE40088	AccI	
Inter-*CND06410*–*CND06420*	42		1779649	JOHE40105	JOHE40106	Hpy188I	

1 : The marker names and the locations are based on JEC21 chromosome 4 (GenBank: NC_006686.1). “Inter-” in the marker name indicates the marker is located in the intergenic region between the two genes in the marker name. Locations of the markers were estimated using the midpoints of the genes, or the intergenic regions between two genes, located on JEC21 chromosome 4.

2 : Markers *SXI1*Dα and *SXI2*D**a** target different alleles of the same locus of the two parental strains that underwent **a**-α bisexual reproduction.

3 : *SXI1*Dα and *SXI2*D**a** are PCR markers. For α-α unisexual production, marker *SXI1*Dα produces PCR products that show size dimorphism between parental strains 431α and XL280αSS due to the C-terminal truncation of the *SXI1*Dα gene in strain 431α [18], and thus, the marker is co-dominant. For **a**-α bisexual reproduction, markers *SXI1*Dα and *SXI2*D**a** are both dominant as they amplify PCR products from parental strains 431α and XL280**a**, respectively.

4 : Markers *SXI2D* **a** and Inter-*CND06040*–*CND06050* were only used for genotyping progeny from **a**-α bisexual reproduction.

5 : *SXI2*D**a** is specific for the *MAT* **a** allele and is not present in JEC21.

**Figure 3:**
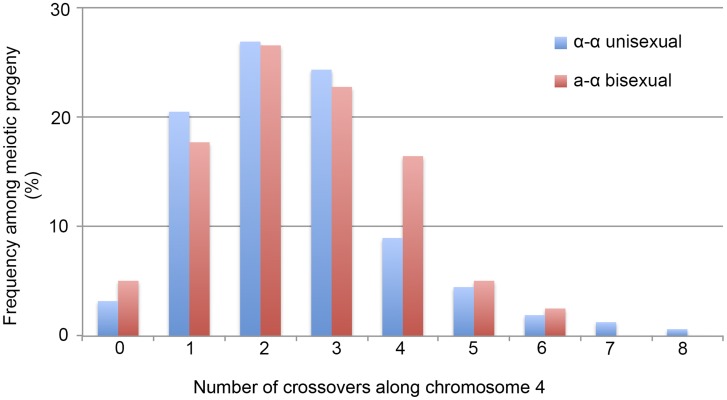
Crossovers are distributed along chromosome 4 during α-α unisexual and a-α bisexual reproduction. Blue bars represent the number of progeny from α-α unisexual reproduction between strains 431α and XL280αSS, while the red bars represent the number of genotypes from **a**-α bisexual reproduction between strains 431α and XL280**a**. The 12 progeny from α-α unisexual reproduction, as well as the 2 genotypes from **a**-α bisexual reproduction that were disomic for chromosome 4 were excluded.

We applied 42 of the 44 genetic markers to genotype the 156 mating products collected from α-α unisexual reproduction. Based on these 42 markers, we identified a total of 74 different genotypes among the 156 mating products recovered from α-α unisexual reproduction. Eleven of the 74 genotypes that were represented by 12 progeny had loci that were heterozygous, suggesting the chromosome 4 in these progeny might be disomic (see below). The number of progeny representing each genotype ranged from 1 to 17, with the majority of the genotypes (66, 89%) being represented by 1 to 3 progeny each. There were two genotypes, No. 52 and No. 60, which were represented by 13 and 17 progeny, respectively (S3 Table). The numbers of detected crossovers along chromosome 4 also varied among the progeny. Of the 144 progeny that were not heterozygous at any of the 42 markers, the majority (126, 87.5%) had 1 to 4 crossovers along chromosome 4 (S4 Table; Fig. 3). The maximum number of crossovers along chromosome 4 was 8, which occurred in one progeny, SSB385 (genotype 23; S3 Table).

To genotype the basidiospores dissected from an individual basidium generated by **a**-α bisexual reproduction, we applied 18 of these 44 genetic markers that corresponded to 17 loci (*SXI1*Dα and *SXI2*D**a** target the same genomic position) (Table 2). In total, we identified 79 genotypes among the 261 basidiospores germinated from 27 basidia (S4 Table). Of the 27 basidia, 26 had spores corresponding to 1 to 4 genotypes, consistent with a single meiotic event in each. For the remaining anomalous basidium (No. 5), we identified 8 different genotypes among the 24 germinated spores (S4 Table). Two genotypes, corresponding to four progeny from 2 (or 3) different basidia, contain one or more loci that were heterozygous, indicating chromosome 4 was likely disomic in these progeny. Among the 79 genotypes that did not contain heterozygous loci, 66 (83.5%) had 1 to 4 crossovers along chromosome 4 (S5 Table). The maximum number of crossovers detected along chromosome 4 was 6, which was observed in two genotypes from basidium 4 (progeny SSB886; S4 Table) and basidium 26 (progeny SSC271; S4 Table), respectively. Interestingly, among all 29 of the basidia dissected from **a**-α bisexual reproduction, other than basidia 8 and 23, from which no spores germinated, basidia 4 and 26 had the lowest spore germination rates at 15% and 12%, respectively (Table 1).

We found that in 5 progeny from α-α unisexual reproduction and 4 progeny from **a**-α bisexual reproduction there was no evidence of recombination along chromosome 4. However, it is still possible that recombination did occur along chromosome 4 in these progeny, but in regions not covered by the genetic markers analyzed, such as subtelomeric regions.

Taken together, our results show clear evidence that recombination occurs along chromosome 4 during α-α unisexual reproduction. Compared to **a**-α bisexual reproduction, crossovers happen at a similar frequency along chromosome 4 (Kolmogorov-Smirnov Test, P>0.05), with the majority of the meiotic products having anywhere between 1 and 4 crossovers.

### Recombination frequencies during α-α unisexual and a-α bisexual reproductions are comparable

**Figure 4:**
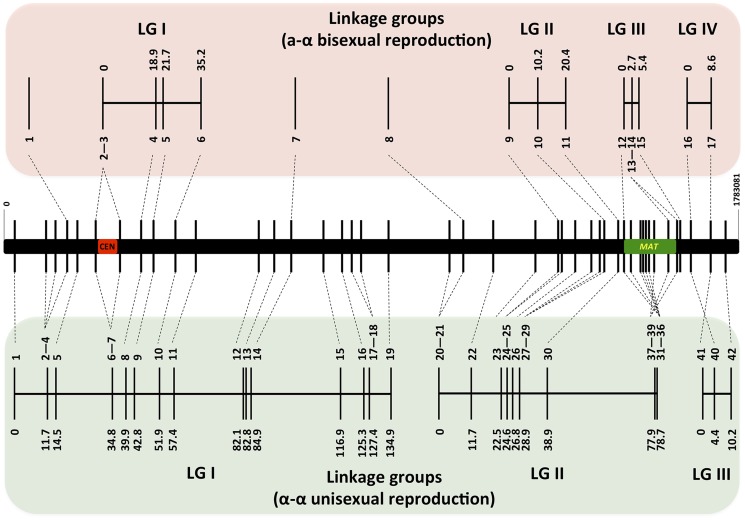
Genetic maps based on analysis of α-α unisexual and a-α bisexual reproduction meiotic progeny. Top and bottom panels: Genetic maps were constructed for **a**-α bisexual and α-α unisexual reproduction, respectively. The genetic map from **a**-α bisexual reproduction contains four linkage groups (LG) and three genetic markers that are not linked to any other markers (Top), while the genetic map from α-α unisexual reproduction contains three linkage groups (Bottom). Numbers at the bottom of linkage groups indicate the serial numbers of the genetic markers analyzed in this study (see Table 2). Numbers at the top of the linkage groups indicate the genetic distance of the markers from the beginning of their respective linkage groups. Middle panel: A schematic illustration of the genetic markers employed to genotype mating products from α-α unisexual reproduction and **a**-α bisexual reproduction. The black bar represents chromosome 4, the vertical lines indicate physical positions of the genetic markers along chromosome 4, and the red and green bars indicate locations of the centromere and *MAT* locus, respectively.

For α-α unisexual reproduction, we constructed a genetic linkage map of the 42 markers based on the genotyping results for the 144 mating products that were monomorphic at all of the 42 genetic markers analyzed. The 42 markers formed three linkage groups (LGI–LGIII) that were 134.9 cM, 78.7 cM, and 10.2 cM in size, and spanned 19, 20, and 3 markers, respectively (Fig. 4). The orders of markers within the linkage groups are in overall agreement with their physical positions on chromosome 4, with the exceptions of three markers (No. 37–No. 39) in LGII and one marker (No. 40) in LGIII (Fig. 4). The three linkage groups had a total length of 223.8 cM, and encompassed 1596 kb of chromosome 4, which produced an average recombination frequency of 7.13 kb/cM (Table 3).

**Table 3:** Recombination frequencies during α-α unisexual and a-α bisexual reproduction.

Interval	Marker 1	Marker 2	Genetic distance (cM)^1^	Physical distance (kb)	Recombination frequency (kb/cM)
**<LGI>**					
1/2	Inter-*CND00070*–*CND00080*	Inter-*CND00310*–*CND00320*	11.7	75	6.41
2/3	Inter-*CND00310*–*CND00320*	Inter-*CND00400*–*CND00410*	0	24	>240^2^
3/4	Inter-*CND00400*–*CND00410*	Inter-*CND00490*–*CND00500*	0	27	>270^2^
4/5	Inter-*CND00490*–*CND00500*	Inter-*CND00610*–*CND00620*	2.9	26	8.97
5/6	Inter-*CND00610*–*CND00620*	Chrom4_220 kb	20.3	47	**2.32** 3
6/7	Chrom4_220 kb	Chrom4_280 kb	0	60	>600
7/8	Chrom4_280 kb	Inter-*CND01130*–*CND01140*	5.1 [19] 1	51	10
8/9	Inter-*CND01130*–*CND01140*	Inter-*CND01270*–*CND01280*	2.9 [2.7]^1^	31	10.69
9/10	Inter-*CND01270*–*CND01280*	Inter-*CND01480*–*CND01490*	9.1 [13.5]^1^	54	5.93
10/11	Inter-*CND01480*–*CND01490*	Inter-*CND01730*–*CND01740*	5.4	52	9.63
11/12	Inter-*CND01730*–*CND01740*	Inter-*CND02320*–*CND02330*	24.7	155	6.28
12/13	Inter-*CND02320*–*CND02330*	Inter-*CND02440*–*CND02450*	0.7	39	55.71
13/14	Inter-*CND02440*–*CND02450*	*CND02580*	2.1	42	20
14/15	*CND02580*	Inter-*CND02890*–*CND02900*	32	81	**2.53** 3
15/16	Inter-*CND02890*–*CND02900*	Inter-*CND03060*–*CND03070*	8.3	45	5.42
16/17	Inter-*CND03060*–*CND03070*	Inter-*CND03160*–*CND03170*	2.1	25	11.90
17/18	Inter-*CND03160*–*CND03170*	Inter-*CND03240*–*CND03250*	0	24	>240^2^
18/19	Inter-*CND03240*–*CND03250*	*CND03480*	7.5	66	8.8
**<LGII>**					
20/21	*CND03960*	Inter-*CND04040*–*CND04050*	0	32	>320^2^
21/22	Inter-*CND04040*–*CND04050*	*CND04340*	11.7	74	6.32
22/23	*CND04340*	*CND04760*	10.8	104	9.63
23/24	*CND04760*	Inter-*CND04980*–*CND04990*	2.2	59	26.82
24/25	Inter-*CND04980*–*CND04990*	Inter-*CND05010*–*CND05020*	0	7	>70^2^
25/26	Inter-*CND05010*–*CND05020*	*CND05140*	2.2	35	15.91
26/27	*CND05140*	*CND05310*	2.2	40	18.18
27/28	*CND05310*	Inter-*CND05360*–*CND05370*	0	22	>220^2^
28/29	Inter-*CND05360*–*CND05370*	Inter-*CND05440*–*CND05450*	0	11	>110^2^
29/30	Inter-*CND05440*–*CND05450*	Inter-*CND05590*–*CND05600*	10 [10.2]^1^	33	3.3^3^
30/31	Inter-*CND05590*–*CND05600*	Inter-*BSP3*-*IKS1*	39.7	15	0.38^3^
31/32	Inter-*BSP3*-*IKS1*	*ZNF1*	0	17	>170^2^
32/33	*ZNF1*	Inter-*STE20*-*MYO2*	0	25	>250^2^
33/34	Inter-*STE20*-*MYO2*	Inter-*LTRTcn10*-*LTRCnirt3*	0	5	>50^2^
34/35	Inter-*LTRTcn10*-*LTRCnirt3*	Inter-*STE3*-*LTRTcn10*	0	1	>10^2^
35/36	Inter-*STE3*-*LTRTcn10*	Inter-*RPO41*-*STE12*	0	7	>70^2^
36/37	Inter-*RPO41*-*STE12*	Inter-*RUM1*-*GEF1*	n.a.^4^	19	n.a.^4^
37/38	Inter-*RUM1*-*GEF1*	*SXI1*Dα	0	35	>350^2^
38/39	*SXI1*Dα	Inter-*CND06020*–*CND06030*	0	24	>240^2^
**<LGIII>**					
40/41	Inter-*CND06130*–*CND06140*	Inter-*CND06290*–*CND06300*	4.4 [8.5]^1^	49	11.14
41/42	Inter-*CND06290*–*CND06300*	Inter-*CND06410*–*CND06420*	n.a.^4^	37	n.a.^4^
			**Total**: 223.8	**Total**: 1596	**Average**: 7.13

1 : Numbers within square brackets are genetic distances in the same intervals during **a**-α bisexual reproduction.

2 : The minimum recombination frequency (kb/cM) within the interval was calculated using 0.1 cM as the estimated maximum genetic distance.

3 : Marker intervals that showed significantly higher recombination frequencies compared to chromosomal intervals within the same LG as well as the chromosomal average.

4 : Could not be inferred due to discrepancies between marker orders within linkage groups and their relative physical location on chromosome 4.

Among the 37 marker intervals that were not interrupted by linkage group breakage or translocation of markers within the linkage groups, genetic distances could be calculated for 22 of them. Of the 15 marker intervals where no genetic distance was detected, one encompasses the centromere of chromosome 4 (LGI, interval 6/7) and 6 are located within the *MAT* locus (LGII, intervals 31/32–37/38) (Table 3), consistent with previous studies suggesting centromeres and the *MAT* locus are typically recombination cold spots [13], [28].

On the contrary, there were also marker intervals that exhibited significantly higher recombination frequencies when compared to other marker intervals, as well as the chromosomal average. Specifically, in linkage group I, two marker intervals, 5/6 and 14/15, had recombination frequencies of 2.32 and 2.53 kb/cM, equal to about 3.1 and 2.9 times the chromosomal average, respectively (Table 3). Interestingly, the interval 5/6 encompasses a region that flanks the centromere of chromosome 4, suggesting that an elevated recombination frequency might be present at the periphery of the centromere. In linkage group II, there were also two marker intervals, 29/30 and 30/31 that showed elevated recombination frequencies at 3.3 and 0.38 kb/cM, equivalent to about 2.1 and 18.9 times the chromosomal average, respectively. Additionally, the recombination frequency in marker interval 30/31 was identified as an outlier when compared with the recombination frequencies from other marker intervals (generalized ESD test), suggesting a significantly higher recombination frequency in this region. Not surprisingly, intervals 29/30 and 30/31 encompass a previously identified recombination hot spot that flanks the *MAT* locus [11].

There were two gaps between the three linkage groups. The gap between LGI and LGII is relatively large and about 152 kb in size. Thus, the lack of genetic markers within this relatively large chromosomal region could be the reason why linkage was not established between LGI and LGII. The gap between LGII and LGIII is relatively small and is only about 31 kb in size. However, this gap encompasses another recombination hot spot that has been previously identified that is flanking the *MAT* locus [11]. This suggests that the same recombination hot spot is likely also operating during α-α unisexual reproduction and the resulting high recombination frequency prevented the establishment of linkage between LGII and LGIII.

For **a**-α bisexual reproduction, we also constructed a linkage map of the 17 loci that were genotyped using the 77 unique genotypes (the two heterozygous genotypes were excluded) that we identified among progeny from the 27 basidia that were dissected. Fourteen of the 17 loci formed four linkage groups (LGI–LGIV; Fig. 4), while no linkage with any other marker was established for the remaining three markers (No. 1, No. 7, and No. 8; Fig. 4). No discrepancy was observed between order of loci within linkage groups and their physical locations on chromosome 4. Similar to α-α unisexual reproduction, there was no genetic distance between the two markers flanking the centromere, as well as among markers located within the *MAT* locus (Fig. 4). The four linkage groups had a total genetic distance of 69.6 cM and encompassed about 529 kb of chromosome 4, producing an average recombination frequency at 7.6 kb/cM that is similar to that observed in α-α unisexual reproduction (7.13 kb/cM; Table 3). Additionally, among the five marker intervals for which genetic distances were calculated for both α-α unisexual and **a**-α bisexual reproduction, four intervals had a trend towards larger genetic distances during **a**-α bisexual reproduction and one interval had a greater genetic distance during α-α unisexual reproduction (Table 3). For two intervals, 7/8 and 40/41 (Table 3), the genetic distances during **a**-α bisexual reproduction were almost 4 and 2 times more than those during α-α unisexual reproduction. However, no statistically significant difference was detected between the genetic distances observed in α-α unisexual and **a**-α bisexual reproduction (Table 3).

Thus, our results support that recombination is occurring at comparable frequencies during α-α unisexual and **a**-α unisexual reproduction in *C. neoformans*. Additionally, the recombination hot spots (e.g. GC rich regions flanking the *MAT* locus) and cold regions (such as within the centromere and the *MAT* locus) that have been identified during **a**-α bisexual reproduction are also operating during α-α unisexual reproduction. Taken together, our results suggest that meiotic recombination is occurring in a similar fashion during the two modes of sexual reproduction.

### Crossover within the *MAT* locus occurs during α-α unisexual reproduction

During **a**-α bisexual reproduction, crossovers are repressed within the *MAT* locus, likely due to the elevated sequence divergence and extensive chromosomal rearrangements that are present between the *MAT* **a** and *MAT*α alleles. However, these physical constraints on meiotic crossover do not exist in α-α unisexual reproduction, and it is not yet clear whether this will result in the *MAT* locus undergoing similar meiotic recombination as other chromosome regions during α-α unisexual reproduction.

**Figure 5:**
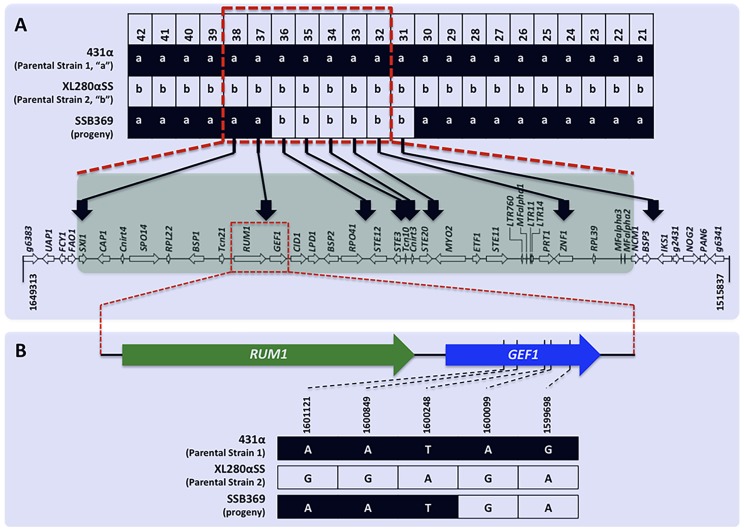
Recombination within the *MAT* locus during α-α unisexual reproduction. A) Of the 42 genetic markers (only No. 21 to No. 42 are shown here for simplicity) that were analyzed for progeny from α-α unisexual reproduction, progeny SSB369 inherited “a” alleles from parental strain 431α for all but 6 markers (No. 31–No. 36; see Table 2 for detailed marker information), which were continuous and located either within or at the edge of the *MAT* locus. The run of allele “b” stopped between markers No. 36 and No. 37 within the *MAT* locus at the 5′ end, and between markers No. 30 and No. 31 that flank the *MAT* locus at the 3′ end. The red dashed-lined rectangle indicates the *MAT* locus. B) Fine mapping conducted by sequencing the region between markers No. 36 and No. 37 showed that in progeny SSB369 the breakpoint within the *MAT* locus for the run of “b” alleles is located inside the *GEF1* gene, within a region of 150 bp in size.

Among the 156 meiotic progeny recovered from α-α unisexual reproduction, we found evidence of crossover within the *MAT* locus in one progeny, SSB369. Specifically, progeny SSB369 inherited alleles from parental strain 431α (“a”) at all but 6 of the 42 genetic markers that were applied for genotyping progeny from α-α unisexual reproduction (S3 Table; Fig. 5). Interestingly, the 6 genetic markers for which progeny SSB369 inherited “b” alleles from parental strain XL280αSS are continuous and are all located within (5 markers) or at the edge (1 marker) of the *MAT* locus. Thus, the *MAT* locus of progeny SSB369 is composed of two tracks of alleles inherited from different parental strains, with alleles from parental strain 431α (“a”) at 2 loci and alleles from parental strain XL280αSS (“b”) at the other 5 loci, with the breakpoint located between markers “Inter-*RPO41*-*STE12*” (No. 36 in Table 2) and “Inter-*RUM1*-*GEF1*” (No. 37 in Table 2) (S3 Table; Fig. 5). There are two possible explanations for this observed pattern. It could be the result of a single gene conversion event encompassing the 6 loci for which progeny inherited the “b” alleles. Alternatively, it could have resulted from two crossover events that are located between markers “Inter-*RPO41*-*STE12*” and “Inter-*RUM1*-*GEF1*”, and between markers Inter-*CND05590*–*CND05600* (No. 30 in Table 2) and Inter-*BSP3*-*IKS1* (No. 31 in Table 2), respectively. By sequencing the region between these two markers from progeny SSB369, as well as the two parental strains, we mapped the breakpoint to an interval of 150 bp in size (between bp 1,600,099 and 1,600,248; Fig. 5) that is located within the *GEF1* gene (Fig. 5). Thus, the run of alleles from parental strain XL280αSS (“b”) within the *MAT* locus of progeny SSB369 is more than 50 kb in size, which is likely too large to be the result of a single gene conversion event. Instead, the mosaic allele composition of the *MAT* locus in progeny SSB369 can be best explained as the result of a crossover event that occurred within the *GEF1* gene, accompanied by another crossover event between markers No. 30 (Inter-*CND05590*–*CND05600*) and No. 31 (Inter-*BSP3*-*IKS1*) that flank the *MAT* locus (Fig. 5).

### Diploid and aneuploid progeny are produced during sexual reproduction

**Figure 6:**
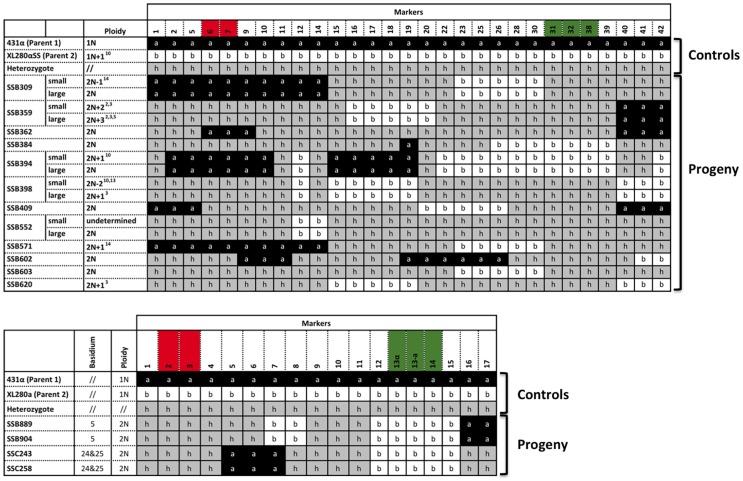
Meiotic progeny disomic for chromosome 4 are produced by α-α unisexual and a-α bisexual reproduction. Top panel: Meiotic progeny from α-α unisexual reproduction between strains 431α and XL280αSS that are disomic for chromosome 4 are shown. Bottom panel: Meiotic progeny from the **a**-α bisexual reproduction between strains 431α and XL280**a** that are disomic for chromosome 4 are depicted. The marker numbers in each panel correspond to those listed in Table 2. For simplicity, not all of the markers for α-α unisexual reproduction are shown (please see S3 Table for complete genotyping profiles). The markers located within the *MAT* locus are highlighted in green, while the markers flanking the centromere are highlighted in red. The values for ploidy were estimated based on FACS analyses and whole genome sequencing (see S1, S2 Figure), where 1N represents haploid and 2N represents diploid. Additionally, the “+” and “−” following 1N or 2N indicate the presence and absence of specific chromosomes, respectively. For example: 2N+2^2,3^ (SSB359-small) represents diploid (2N) with additional two chromosomes (ch. 2 and 3). The small colony from progeny SSB552 appears to be a mixture of individuals with varying ploidy levels (S2 Figure), and is thus designated as “undetermined” here.

Among the 156 progeny that we recovered from α-α unisexual reproduction, 12 (7.7%; or 14.9% if only unique genotypes are considered) were heterozygous for multiple genetic markers that we employed for genotyping, and the number of heterozygous loci ranging from 8 to 39 (Fig. 6). Similarly, among the 27 basidia that were dissected from **a**-α bisexual reproduction, we found 4 progeny from two separate basidia (7.4%; or 3.7% is all unique genotypes are considered), basidium No. 5 (progeny SSB889 and SSB904) and basidium No. 24 & 25 (progeny SSC243 and SSC258), that were heterozygous for 8 and 9 of the 17 loci that were analyzed, respectively (Fig. 6). All of these progeny were uninucleate based on DAPI staining, and FACS analyses showed that the nuclei of these progeny had about twice the DNA content as the haploid parental strains (S1 Figure), suggesting these progeny are diploid.

To confirm the ploidy of these meiotic progeny containing two copies of chromosome 4, genomes were sequenced for each of the 12 progeny from α-α unisexual reproduction and two progeny representing the two different genotypes from **a**-α bisexual reproduction. Read depths were calculated for each chromosome of each progeny. In addition, among the 12 meiotic progeny from α-α unisexual reproduction, we found 5 progeny that each produced distinctly large and small colonies when grown on YPD solid medium (Fig. 6 and Fig. 1C). For each of these 5 progeny, both large and small colony isolates were subjected to whole genome analysis.

**Figure 7:**
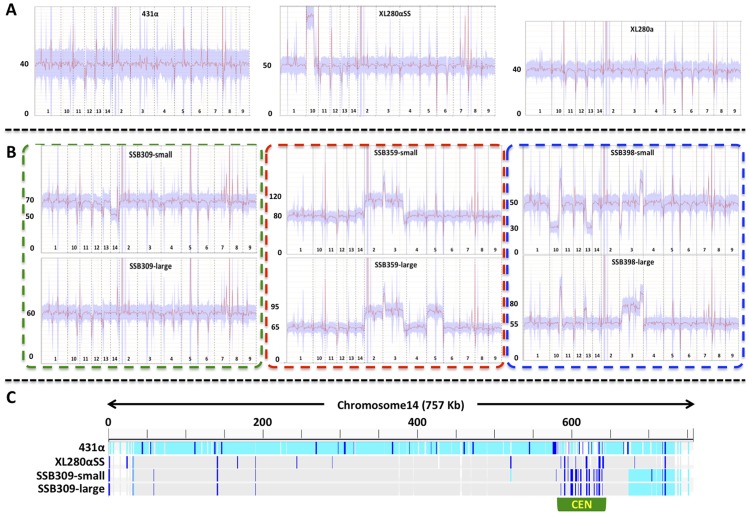
Transient aneuploid and persistent diploid progeny are generated from α-α unisexual reproduction. A) The three panels show the read depths of all chromosomes in the three parental strains: 431α, XL280αSS, and XL280**a**. For each panel, the x-axis indicates the 14 chromosomes and the y-axis indicates the read depth from the Illumina sequencing. These strains had only one copy of chromosome 4, as no heterozygosity was detected for any marker from chromosome 4 that was analyzed, and thus, all are haploid. This was also consistent with results from the FACS analyses (S1 Figure). B) The six panels show the read depths of all chromosomes in strains derived from three meiotic progeny from α-α unisexual reproduction between strains 431α and XL280αSS. For each panel, the x-axis indicates the 14 chromosomes and the y-axis indicates the read depth from the Illumina sequencing. All of these strains showed heterozygosity at multiple markers along chromosome 4 (Fig. 6), indicating they had at least two copies of chromosome 4. FACS analyses indicated that these strains were likely diploid (S1 Figure), which was consistent with the sequencing results shown here suggesting all of the meiotic progeny had two copies for the majority of the chromosomes (including chromosome 4). The two panels within each of the three dashed-lined rectangles are sequencing results of small and large colonies, respectively, that were derived from the same meiotic progeny. C) The distribution of SNPs along chromosome 14 in the small and large colonies of the progeny SSB309, as well as the two parental strains 431α and XL280αSS. The genome sequence of each isolate was mapped against the published genome sequence of strain JEC21. The light blue bars indicate SNPs compared to JEC21, dark blue bars indicate heterozygous sites, while grey areas indicate regions that are identical to JEC21. The centromeric region of chromosome 14 is highlighted by the green rectangle.

Because all of the progeny with two copies of chromosome 4 were shown to be diploid (or aneuploid) based on FACS analysis, we assigned the copy number of chromosome 4 as two for each of the progeny. Accordingly, we found that for the two meiotic progeny from **a**-α bisexual reproduction, the copy numbers of each of their chromosomes were the same as that of chromosome 4. Thus they were diploid and euploid (Fig. 7 and S2 Figure). For the 12 meiotic progeny from α-α unisexual reproduction, we found 5 of the 7 progeny that did not produce large and small colonies were diploid and euploid, while the other two, SSB571 and SSB620, were aneuploid. Specifically, progeny SSB571 had two copies of all chromosomes except chromosome 14, which was trisomic with three copies (2N+1; S2i Figure). Progeny SSB620 had two copies of all chromosomes except chromosome 3, which was trisomic (three copies) for the majority of chromosome 3, and disomic (two copies) and tetrasomic (four copies) for the two ends of chromosome 3, respectively (S2j Figure).

For the 5 meiotic progeny from α-α unisexual reproduction that produced large and small size colonies (Fig. 6), we found that at least one type of colony for each was aneuploid based on genome sequencing (Fig. 7 and S2 Figure). Specifically, for three progeny (SSB309, SSB394, and SSB552), the large colonies were diploid and euploid while the small colonies were aneuploid, and for two progeny (SSB359 and SSB398) both large and small colonies were aneuploid (Fig. 7 and S2 Figure). Our results also indicated that chromosomal gain/loss had occurred between the large and small colonies from the same meiotic progeny. For example, genome sequencing and FACS analysis showed that the large colony (SSB309-large) of progeny SSB309 was diploid and euploid, while the small colony (SSB309-small) had a ploidy of 2N-1 with only one copy of chromosome 14 (Fig. 7B). By analyzing the SNPs between the two parental strains, we found that the only copy of chromosome 14 in SSB309-small was recombinant, and the two copies of the chromosome 14 in SSB309-large were nearly identical and the same as chromosome 14 in SSB309-small (Fig. 7C). Thus, SSB309-large (2N) likely has evolved from SSB309-small (2N-1) by acquiring an extra copy of chromosome 14 to return to euploidy with loss of heterozygosity due to chromosome loss and duplication of the remaining chromosome. Similarly, for progeny SSB398, the small colony (SSB398-small) has one copy, and the large colony (SSB398-large) has two copies, for both chromosomes 10 and 13, respectively (Fig. 7). Again, analyses based on SNPs between the two parental strains showed that the two copies of both chromosome 10 and 13 in SSB398-large were nearly identical and the same as the single copy in SSB398-small (except at the regions where SSB398-large has ploidy higher than 2N, which could be due to segmental duplication and/or chromosomal translocation; S2, S3 Figure), suggesting that the small colony is ancestral and the large colony evolved through acquisition of extra copies of chromosomes. Additionally, chromosome 3 of the large and small colonies of progeny SSB394, and chromosome 11 of the large and small colonies of progeny SSB359 also appear to be homozygous along the whole chromosome, suggesting that these chromosomes have also undergone chromosomal loss and duplication that result in loss of heterozygosity of the whole chromosome, similar to what have been observed in chromosome 14 of the progeny SSB398 (S2, S3 Figure).

Because we dissected individual basidiospores from **a**-α bisexual reproduction to collect meiotic progeny, the two diploid genotypes from **a**-α bisexual reproduction were due to either 1) packaging of two euploid haploid meiotic products into one spore during sexual reproduction with subsequent karyogamy or 2) fusion of two post-meiotic nuclei and packaging of a diploid nucleus into the spore. However, for the 12 diploid and aneuploid genotypes from α-α unisexual reproduction, they could be either due to packaging two haploid nuclei or one diploid nucleus into one spore during sexual reproduction, or they could be the result of secondary fusion of two F1 progeny during our screening process (see also Discussion below).

### Biased allele inheritance in progeny from α-α unisexual and a-α bisexual reproduction

**Figure 8:**
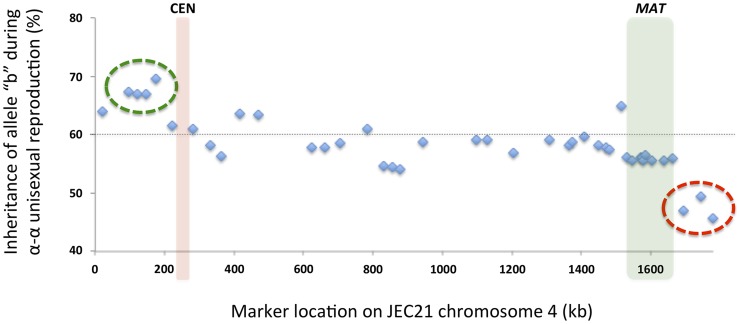
Biased allele inheritance among progeny from α-α unisexual reproduction. The X-axis shows the positions of the markers, and the Y-axis indicates the percentage (%) of unisexual progeny that inherited allele “b” from parental strain XL280αSS at each marker. The dashed line indicates 60%. The green circle highlights a cluster of four markers that showed particularly high allele “b” frequencies, while the red circle highlights a cluster of three markers that showed relatively lower allele “b” frequencies. The red and green columns indicate the locations of the centromere and *MAT* locus, respectively.

Among the progeny recovered from α-α unisexual reproduction, we observed a general bias toward inheriting “b” alleles from the parental strain XL280αSS (Fig. 8). Specifically, across all 42 markers, the frequencies of allele “b” among the progeny from α-α unisexual reproduction ranged between 45.7% and 69.6%, with an average of 58.3%. Interestingly, this inheritance bias toward “b” alleles was most significant in a cluster of 4 markers located close to the left end of chromosome 4 (green circle in Fig. 8). In contrast, a cluster of 3 markers located on the opposite end of chromosome 4 showed a significant allele inheritance bias toward the “a” alleles from parental strain 431α (red circle in Fig. 8).

**Figure 9:**
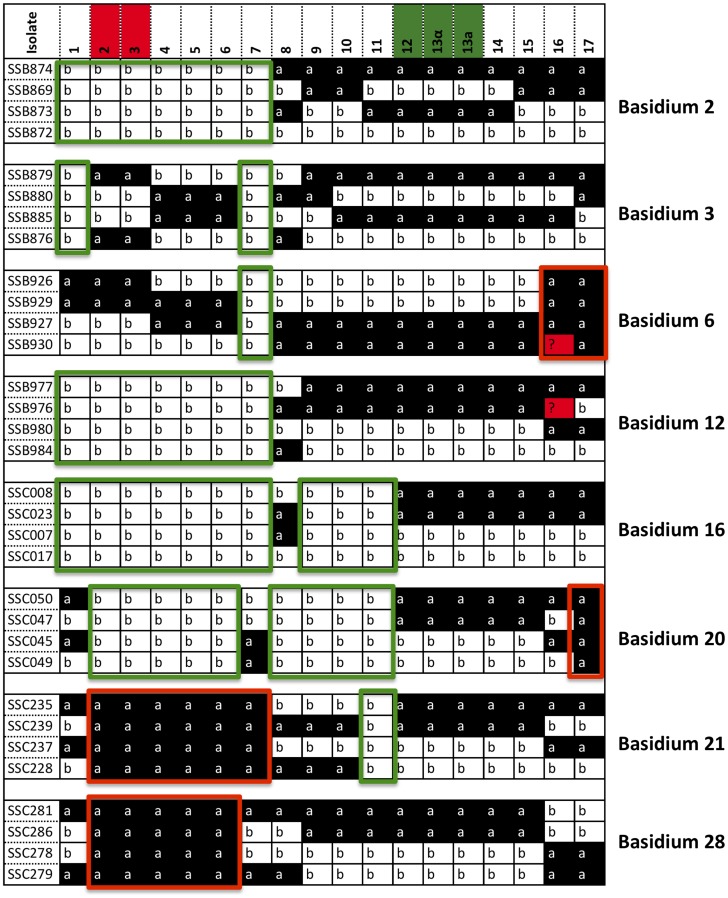
Biased allele inheritance among progeny from a-α bisexual reproduction. Numbers in the top row indicate the loci/genetic markers used for genotyping progeny from **a**-α bisexual reproduction, with the markers flanking the centromere are highlighted in red and the markers within the *MAT* locus are highlighted in green (see Table 2 for detailed information). For simplicity, only the unique genotypes are shown for each of the eight basidia that showed biased allele inheritance. The markers that exhibited biased allele inheritance are highlighted by rectangles, with red color indicating a bias toward the “a” allele and green color indicating a bias toward the “b” allele.

Similarly, we also found evidence of biased allele inheritance among progeny that were dissected from **a**-α bisexual reproduction. Specifically, of the 26 basidia from which viable spores were recovered (basidium No. 15 was excluded because only one viable spore was recovered), for 8 basidia (30.8%) there was at least one (and up to 10) genetic marker(s) for which the allele from one of the two parental strains was absent among all viable spores (Fig. 9). All eight basidia generated spores that belonged to four different genotypes, and the germination rates of the spores from these eight basidia were higher than 80% except for two basidia, No. 3 and No. 20, that had spore germination rates of 50% and 46%, respectively. Among these eight basidia, the absence of an allele from one of the two parental strains occurred in 13 of the 17 loci analyzed. Interestingly, of the four loci for which no biased allele inheritance has been observed during **a**-α bisexual reproduction, one locus, *SXI1*Dα/*SXI2*D**a**, is located within the *MAT* locus, while the other three, Inter-*BSP3*-*IKS1*, Inter-*CND06020*–*CND06030* and Inter-*CND06030*–*CN06040*, are located within regions that flank the *MAT* locus (Table 2; Fig. 9).

Of the cluster of four genes that showed the most significant biased inheritance towards “b” alleles during α-α unisexual reproduction, marker Inter-*CND00490*–*CND00500* was also used for genotyping progeny from **a**-α bisexual reproduction. Allele “b” of marker Inter-*CND00490*–*CND00500* was present in progeny from all of the 26 basidia dissected from bisexual reproduction. However, in four of the 26 basidia (basidia 2, 12, and 16; Fig. 9) the “a” allele from parental strain 431α for this marker was absent in all of the progeny that germinated, which is consistent with the biased inheritance toward the “b” allele observed at this marker during α-α unisexual reproduction.

Of the cluster of three genes that showed the most significant biased inheritance towards “a” alleles during α-α unisexual reproduction, markers Inter-*CND06130*–*CND06140* and Inter-*CND06290*–*CND06300* were also employed to genotype progeny from **a**-α bisexual reproduction. The “a” alleles for both markers were present in progeny from all of the 26 basidia dissected. However, the “b” alleles for markers Inter-*CND06130*–*CND06140* and Inter-*CND06290*–*CND06300* were absent among progeny from 1 (basidium 6) and 2 (basidia 6 and 20) basidia, respectively (Fig. 9). Again, this is consistent with the more biased inheritance towards “a” alleles at these loci observed in α-α unisexual reproduction.

## Discussion

In *C. neoformans*, a leading human fungal pathogen, mating can be bisexual, occurring between isolates of opposite mating type, *MAT* **a** and *MAT*α. However, it has been recently discovered that sexual reproduction also occurs between isolates of the same mating type, especially *MAT*α [16]. Similar plasticity in mating compatibilities occurs in *C. albicans*, another important human fungal pathogen [24]. The relaxed constraints on mating compatibility and consequently increased chances for sexual reproduction could provide evolutionary advantages for these pathogenic fungi, manifested as changes in ploidy and generation of novel genotypes through recombination [18].

Although unisexual reproduction in *C. neoformans* has been well characterized qualitatively, detailed quantitative assessments of this unique mode of sexual reproduction have not. One outstanding question is the extent of recombination during α-α unisexual reproduction, and how this compares to **a**-α bisexual reproduction. Put another way: Does the level of recombination during α-α unisexual reproduction support its assignment as a complete meiotic sexual cycle? In the study that first described α-α unisexual reproduction in *C. neoformans*, the authors analyzed eight progeny using 20 genetic markers located on 5 chromosomes [16]. They found clear evidence of independent chromosomal segregation among the 5 chromosomes; additionally, recombination was detected among markers on 4 of the 5 chromosomes [16]. However, the genetic markers were widely dispersed along each chromosome, and the number of progeny analyzed was relatively small, which prevented a more quantitative assessment of recombination frequencies during α-α unisexual reproduction.

In our study, we found that the two modes of sexual reproduction resulted in similar phenotypic segregation among meiotic progeny, including transgressive phenotypes observed under a variety conditions (Fig. 1). Additionally, frequencies of crossovers along chromosome 4 in the two modes of sexual reproduction were comparable (S5 Table and Fig. 3). Furthermore, of the five chromosomal regions for which we were able to calculate genetic distances for both α-α unisexual and **a**-α bisexual reproduction, four showed comparable genetic distances with less than twofold differences between the two modes of sexual reproduction (intervals 8/9, 9/10, 29/30, and 40/41 in Table 3), while the other interval had genetic distances that are ∼4 times higher in **a**-α bisexual reproduction than in α-α unisexual reproduction (intervals 7/8 in Table 3). These results provide definitive support that α-α unisexual and **a**-α bisexual reproduction involve similar meiotic processes.

The average recombination frequency observed during α-α unisexual reproduction was ∼7.13 kb/cM. This is higher than the genome average of ∼13.2 kb/cM previously reported for **a**-α bisexual reproduction in serotype D *C. neoformans* [13]. There are several reasons why we may have observed a higher overall recombination frequency occurring along chromosome 4. First, as discussed below, chromosome 4 contains two recombination hot spots that flank the *MAT* locus, which could elevate the average recombination frequency of chromosome 4 compared to the genome wide average, even after taking into account that recombination is repressed within *MAT* (Fig. 4). Additionally, in the previous Marra et al. study, although linkage groups were established for most of the genome, some chromosomal regions were not included in the linkage map [13]. Thus, the genome wide recombination frequency average could be an underestimate. For example, there were two linkage groups assigned to chromosome 4 [13], which correspond to LGII and LGIII in our α-α unisexual reproduction linkage map (Fig. 4). Thus, a linkage group (LGI; Fig. 4) of about 130 cM in size encompassing a chromosomal region of more than 900 kb (Table 2) was not present in the Marra et al. linkage map [13]. Because the authors used the total genetic map length and genome size to calculate the average recombination frequency, the omission of linkage groups could have underestimated the genome wide recombination frequency.

We also observed that recombination frequencies were not distributed evenly along chromosome 4 during α-α unisexual reproduction, and several possible recombination hot spots were present. First, there were two gaps among the three linkage groups that were established for chromosome 4. The gap between LGI and LGII (between markers No. 19 and No. 20; Table 2 and Fig. 4) was relatively large (152 kb in size), and thus could be due to a paucity of sufficient genetic markers within this region to bridge the two linkage groups. However, the gap between LGII and LGIII encompassed a relatively small chromosomal region (31 kb in size; between markers No. 39 and No. 40). Given the population size of the meiotic progeny that were analyzed, the failure to establish linkage between LGII and LGIII was then most likely due to a recombination hot spot within this region. Notably, this gap overlaps with one of two recombination hot spots flanking *MAT* previously shown to operate during **a**-α reproduction [11]. Also consistent with previous studies, the region flanking the other side of *MAT* also showed elevated recombination frequencies during α-α unisexual reproduction (between markers No. 30 and No. 31; Table 3 and Fig. 4). Specifically, the recombination frequency between markers No. 30 and No. 31 (interval 30/31 in Table 3) was 0.38 kb/cM, more than 18 times the chromosomal average (7.13 kb/cM). Thus, the recombination hot spots that have been previously identified to flank *MAT* and operate during **a**-α bisexual reproduction also function during α-α unisexual reproduction.

Elevated recombination frequencies were also found in two other regions within LGI, intervals 5/6 and 14/15, within which recombination occurred at frequencies that were about 3.1 and 2.9 times the chromosomal average, respectively (Table 3). It is not clear what causes the increased recombination frequency within the interval 14/15. However, for interval 5/6, it is located in the chromosomal region that flanks the centromere of chromosome 4. Centromeres and their flanking regions are classically considered crossover deserts. Indeed, it has been shown that in yeast centromere-proximal crossovers usually lead to precocious separation of sister chromatids during meiosis, and is often responsible for spore inviability [29]. However, recent studies reveal these recombination cold spots might not be that cold after all. For example in *Arabidopsis*, unexpectedly high levels of gene conversion have been observed near centromeres [30]. Additionally, extensive gene conversions were found within centromere cores in maize [31]. Thus, the elevated recombination frequency within interval 5/6 could be due to extensive gene conversion. Interestingly, one of the two markers that flanks interval 5/6, marker No. 5, exhibited the most biased allele inheritance during α-α unisexual reproduction (Table 2 and Fig. 8), which would also be consistent with biased gene conversion being prevalent in this region. The recombination frequency in the chromosomal region flanking the other side of the centromere (interval 7/8 in Table 3) was 10 kb/cM during α-α unisexual reproduction, not significantly different from the chromosomal average. However, the same interval had a recombination frequency that was almost 4 times higher (19 cM and 2.68 kb/cM) during **a**-α bisexual reproduction (Table 3), indicating a recombination hot spot might operate within this region. Although no crossover has been observed within the centromeric region in either α-α or **a**-α reproduction, the lack of suitable genetic markers within the centromere prevented us from investigating if intra-centromeric gene conversion had occurred during sexual reproduction.

No recombination has been detected within *MAT* during **a**-α bisexual reproduction, which is consistent with the notion that recombination within *MAT* is highly repressed during sexual reproduction, presumably due to rearrangements between the *MAT* **a** and *MAT*α alleles. However, gene conversion does occur at high frequency around a GC rich region within *MAT* during **a**-α bisexual mating [12], suggesting that *MAT*, just like centromeres, might not be as bereft of recombination as previously thought. Interestingly, among the 156 mating products and eight genetic markers that were analyzed for α-α unisexual reproduction, one recombination event was detected within *MAT* that likely resulted from crossover. Specifically, the *MAT* locus of progeny SSB369 contained two stretches of alleles inherited from different parents, with the breakpoint located in a region of 150 bp in size within the *GEF1* gene (between nucleotides 1,600,099 and 1,600,248 on chromosome 4; Fig. 5). Although this pattern could be the result of a gene conversion event, the track would have to be >50 kb in size, which is rare. Thus, we hypothesize that the observed *MAT* locus allele composition in progeny SSB369 is the result of a double crossover, with one occurring at the breakpoint within *MAT* and the other at the edge of *MAT* between markers 30 and 31 (Fig. 5).

Given that the alleles of the *MAT* locus are co-linear between the two parental *MAT*α strains, it is thus not surprising that crossover occurred within *MAT* during α-α unisexual reproduction. Instead, given the size of *MAT* (>100 kb) and the chromosomal average recombination frequency (7.13 kb/cM), we would have expected to identify more than 21 events of inter-*MAT* recombination among the 156 α-α unisex progeny analyzed. The lower than expected frequency has two possible explanations. First, repair of double strand breaks (DSBs) induced within *MAT* may have an innate bias toward pathways leading to gene conversions (e.g. Synthesis Dependent Strand Annealing, SDSA) than pathways yielding crossovers (e.g. Double Strand Break Repair, DSBR). Second, we have found that, similar to **a**-α reproduction, recombination hot spots also operate flanking *MAT* during α-α unisexual reproduction. Thus, inter-*MAT* recombination, including crossovers and gene conversions, could have been repressed due to interference exerted by these recombination hot spots even though no chromosomal rearrangements are present within *MAT* during α-α unisexual reproduction. We note that the *GEF1* gene, within which the hypothesized crossover breakpoint is located, lies in a cluster of *MAT* genes (*GEF1* to *RPO41* in Fig. 5A) that show the least divergence between the *MAT* **a** and *MAT*α alleles of *C. neoformans* [32]. Additionally, the intergenic region between the *RPO41* and *BSP2* genes, where a gene conversion hot spot has been identified during **a**-α bisexual reproduction [12], is also located within this gene cluster, and is near the *GEF1* gene (Fig. 5). Thus, the high similarity between **a** and α alleles within this gene cluster could be the result of ongoing recombination (crossovers and gene conversions) in this region during α-α and **a**-α reproduction.

Previous studies have found that diploid/aneuploid progeny can also be readily produced at high frequency during hybridization between serotypes A and D *C. neoformans*, which is likely due the elevated sequence divergence as well as the large number of chromosomal rearrangements that are present between the parental strains [28], [33], [34]. In our study, from α-α unisexual and **a**-α bisexual reproduction, we recovered 12 and 4 progeny, respectively, that were heterozygous for portions of the genetic markers analyzed (Fig. 6). Subsequent FACS analyses and genome sequencing showed that four progeny from **a**-α bisexual reproduction, as well as five of the 12 progeny from α-α unisexual reproduction were diploid (Fig. 6, S1, S2 Figure). Of the other 7 progeny from α-α unisexual reproduction that had two copies of chromosome 4, two produced colonies uniform in size and were shown to be diploid with one additional chromosome (2N+1; SSB571 and SSB620; Fig. 6 and S1, S2 Figure), while the other 5 produced dimorphic (large and small) colonies on YPD plates. By sequencing both large and small colonies from each of these five progeny, we found that for two progeny (SSB359 and SSB398) both large and small colonies were aneuploid (Fig. 6 and 7), while for the other three progeny the large colonies were diploid and the small colonies were aneuploid (Fig. 6 and 7; S1, S2 Figure). Furthermore, our data suggest that for two of these three progeny, the diploid large colonies were derived from the ancestral small aneuploid colonies by chromosome gain.

These diploid/aneuploid progeny were meiotic products, as they contained chromosomes that had clearly undergone recombination (Fig. 6). Thus, they could be the result of either errors that occurred during meiosis that caused chromosomal mis-segregation, or due to packaging of two meiotic products into a single spore during spore generation. Additionally, for the 12 diploid/aneuploid progeny from α-α unisexual reproduction that were disomic for chromosome 4, because they were not isolated by spore dissection, it is possible they result from secondary cell-cell fusion between two meiotic products during the screening process.

We observed a frequency between 7.7% and 14.9% (see Results) for diploid/aneuploid progeny generated by α-α unisexual reproduction, while for **a**-α bisexual reproduction, the frequency of diploid/aneuploid progeny was between 3.7% and 7.4%. In a recent study by Ni and Feretzaki et al. [26], aneuploidy was found to be generated at a frequency between 4% and 5% during both α-α unisexual and **a**-α bisexual reproduction in *C. neoformans*, slightly lower than our observations, which included both diploid and aneuploid progeny. However, in our study, we only analyzed ploidy in detail for progeny disomic for chromosome 4; thus, the frequency observed represents the lower limit of the actual frequency of aneuploid progeny generated during α-α unisexual reproduction. Additionally, we found that for three meiotic progeny from α-α unisexual reproduction that produced dimorphic colonies (SSB309, SSB394, and SSB552), the larger colonies were diploid while the smaller colonies were aneuploid. Thus, aneuploidy could be transient and lost during sub-culturing without a suitable selection pressure, as it can reduce fitness. This could also lead to underestimation of the frequency of aneuploidy generated by sex.

We found that persistent diploid progeny were frequently generated from both α-α unisexual and **a**-α bisexual reproduction in *C. neoformans*. Being able to transit between haploid and diploid could be an evolutionary advantage. For example, in a recent study, a group of *Saccharomyces cerevisiae* and *Saccharomyces paradoxus* isolates were tested for their fitness as either haploids or diploids under a variety of conditions [35]. Interestingly, for the conditions in which significant fitness differences were observed between haploids and diploids, haploids were more fit in half of the conditions, while diploids exhibited higher fitness in the other half of conditions. Additionally, there was a clear correlation between the two species as to which ploidy states had higher fitness under certain conditions. These findings suggest that the ability to transit between the two ploidy states might be beneficial when the environment changes from those favoring haploid to those favoring diploids, or vice versa. Additionally, compared to haploids, diploids may have a higher evolutionary capacity, both from being more tolerant of deleterious mutations and thus allowing the establishment of negative epistatic interactions, and from having twice as much genetic material for evolution to act upon. Furthermore, the transition from haploid to diploid also allows mitotic recombination to occur, which could have significant phenotypic consequences if genetic variation exists between the two progenitor strains. Thus, by enabling rapid ploidy transitions, sexual reproduction confers benefits to organisms even in the absence of shuffling genetic information through meiotic recombination. Such parasexual processes, in which diploids are formed through fusion of haploid nuclei, and ensuing whole chromosome losses, accompanied with mitotic recombination, eventually restore the haploid state, have been observed in many fungal species, such as *C. albicans* and *Aspergillus nidulans*. It has been found in a recent study that in *A. nidulans*, faster growing isolates frequently arose from homozygous diploid strains, but not from the haploid strains from which the homozygous diploid strains were formed [36]. Additionally, the faster growing variants had all reverted to haploidy through parasexual recombination and chromosomal losses.

Among progeny recovered from α-α unisexual reproduction, there was an overall bias toward inheriting alleles from the parental strain XL280αSS (“b” alleles; Fig. 8). Specifically, for all 42 genetic markers, only three showed less than 50% of “b” alleles among the meiotic progeny. Interestingly, these three markers formed a cluster that is located at one end of chromosome 4 (the red circle in Fig. 8). On the other hand, the four genetic markers with the most significant bias towards “b” alleles also formed a cluster that is located in a region flanking the centromere (the green circle in Fig. 8). We observed consistent allele inheritance biases during **a**-α bisexual reproduction. Specifically, the markers that exhibited the most significant biases toward “b” alleles were found to have only “b” alleles inherited in some of the basidia, and vice versa (see Results). These biases could be due to biased gene conversion. However, gene conversion is usually regional, and the conversion tracks are typically short. In most cases where biased allele inheritance was observed during **a**-α bisexual reproduction, the absence of alleles from one of the two parents involved multiple continuous markers that encompassed large chromosomal regions, and thus are less likely due to gene conversion. Additionally, markers exhibiting the most and least biased inheritances formed clusters encompassing relatively large chromosomal regions. This prompted us to hypothesize that the biased allele inheritance patterns are the result of loss of heterozygosity, which was likely caused by mitotic recombination that occurred after nuclear fusion but prior to meiosis. It has been shown recently that the *S. cerevisiae* genome contains fragile sites prone to mitotic recombination that leads to loss of heterozygosity [37]. In *C. neoformans* the two nuclei are typically separate during hyphal growth, and meiosis occurs shortly after nuclear fusion occurs within the basidium. However, nuclear fusion could happen, and has been observed, at earlier stages of sexual development (e.g. in the zygote or hyphae) [38], [39], which could provide opportunities for mitotic recombination to result in loss of heterozygosity at certain chromosomal regions. Interestingly, among meiotic progeny from **a**-α bisexual reproduction, two of the marker intervals (between markers Inter-*CND00490*–*CND00500* and Chrom4_220 kb and between markers *CND02580* and Inter-*CND04040*–*CND04050*) that are most frequently flanking the chromosomal regions within which heterozygosity is lost are both located within chromosome regions where elevated recombination frequencies were observed during α-α unisexual reproduction. Thus, these chromosomal regions may contain recombination hot spots that actively induce recombination during meiosis, and given the opportunity, can also incur frequent mitotic recombination that results in loss of heterozygosity.

In previous studies, it has been shown that the key meiotic regulators of **a**-α bisexual reproduction, the endonuclease Spo11 and the meiotic recombinase Dmc1, are also required for spore production and germination during α-α unisexual reproduction [16], [17], suggesting unisexual reproduction is a meiotic process. By analyzing two progeny populations generated from α-α and **a**-α sexual reproduction, respectively, we showed that the two types of sexual reproduction are indeed occurring in very similar fashions in *C. neoformans*. In both sexual modes, we found that although mitotic recombination does occur, the majority of recombination is likely meiotic. Additionally, several aspects of meiotic recombination are also comparable between unisexual and bisexual reproduction, such as the number of crossovers along the chromosome, the recombination frequencies in various chromosomal regions, as well as the frequencies at which persistent diploids were generated. Our study thus provides substantial evidence that unisexual reproduction in *C. neoformans* involves meiosis just as during bisexual reproduction. The ability to undergo unisexual reproduction in a way that mimics traditional bisexual reproduction could significantly increase opportunities for successful mating in *C. neoformans*. Thus, unisexual reproduction could be a strategy acquired during evolution that allows organisms to take full advantage of sexual reproduction, and to cope with challenging environments.

## Materials and Methods

### Strains employed in this study

For α-α unisexual reproduction, we used strains 431α, which is a natural serotype D, *MAT*α isolate [12], and strain XL280αSS, which is a spontaneous 5-FOA resistant (*ura5*, chromosome 7) XL280α strain, and with a NAT resistant marker ectopically inserted in its genome (chromosome 1). For **a**-α bisexual reproduction, we used strains 431α and XL280**a**. Strain XL280**a** is congenic with XL280αSS, except for the *MAT* locus and the loci where the *ura5* and NAT^R^ markers are located [27].

CHEF gel electrophoresis showed that these three strains have similar karyotypes (S4A Figure), although whole genome sequencing indicated strain XL280αSS has a section of chromosome 10 that is duplicated compared to strain 431α (Fig. 7). Whole genome sequencing also showed that the natural strain 431 has an average sequence similarity of >99.5% for each of the 14 chromosomes compared to the serotype D type strain JEC21 (Sequence Read Archive project accession number: SRP042617). Additionally, using 11 serotype D *MAT* **a** specific and 13 serotype D *MAT*α specific PCR markers (S6 Table), we confirmed that strain XL280**a** possesses the serotype D *MAT* **a** allele, while strains 431α and XL280αSS possess serotype D *MAT*α alleles (S4B–S4C Figure). However, variations are also present between the two *MAT*α alleles from strains 431α and XL280αSS. For example, two of the *MAT*α specific markers (No. 9 and No. 10) produced PCR products that are larger in size from strain XL280αSS than from strain 431α (S4C Figure). Additionally, we discovered that a region of about 5 kb in size, which is located at the edge of the chromosome 4 centromere in XL280αSS and encompasses genetic marker “Chrom4_270 kb” (Table 2), has been translocated in strain 431α. As the result, we found that marker “Chrom4_270K” showed a high level of heterozygosity, or was absent from many meiotic progeny (last column in S3 Table), as the two alleles from strains 431α and XL280αSS are now on separate chromosomes and are segregating independently during meiosis. Consequently, we excluded marker “Chrom4_270 kb” from our linkage analysis.

All strains were maintained in −80°C frozen stocks in 35% glycerol and sub-cultured from freezer stocks to YPD solid medium for study.

### Laboratory crosses and meiotic progeny isolation

Mating was carried out as previously described [40]. Briefly, mating compatible strains were mixed and spotted on V8 (pH = 5) medium. The mating plates were incubated in the dark at room temperature (agar side up with no parafilm) for ∼1 week until abundant hyphae, basidia, and basidiospore chains were visible under the microscope.

For crosses between strains 431α and XL280αSS, the hyphal sectors at the periphery of the mating spots were excised and suspended in 1× PBS. The cell suspension was diluted, and spread onto SD-uracil plates to screen for Ura^+^ isolates. The Ura^+^ isolates were then transferred onto YPD+NAT plates to further screen for isolates that were also NAT resistant, and thus represented recombination/fusion of the markers present in the two parental strains.

For crosses between strains 431α and XL280**a**, spore chains from an individual basidium were transferred onto fresh YPD medium, and individual basidiospores were separated using a fiber optic needle spore dissecting system, as previously described [9]. Individual spores separated from the same basidium are the products of one meiotic event [9].

### Phenotypic analyses of meiotic progeny

We analyzed phenotypic variation among the meiotic progeny under a variety of conditions, including growth on YPD at different temperatures (37, 40, and 41°C) for temperature tolerance, pigmentation on L-DOPA medium plate for melanin production, and hyphal and spore production on MS medium plates for selfing ability.

To phenotype the progeny, cells from 1 ml overnight YPD culture were pelleted by centrifuge, washed twice using water, and finally resuspended in 10 ml water. And 4 µl of the cell suspensions was then spotted onto the media plates for phenotyping. On each phenotyping plate, the two parental strains from which the progeny were isolated were also included as controls.

### Genomic DNA, genetic markers, and genotyping

Germinated individual spores were transferred and patch-streaked onto fresh YPD medium, and genomic DNA was extracted from the biomass as described in a previous study [12]. We developed a total of 44 genetic markers spanning chromosome 4 of *C. neoformans*, of which two were PCR markers (present/absent) and 42 were co-dominant PCR-RFLP markers (i.e. they can differentiate the two homozygous states, as well as the heterozygous one; Table 2; Fig. 2). 42 genetic makers were used to analyze progeny from α-α unisexual reproduction between strains 431α and XL280αSS, while 18 genetic markers were applied to genotype progeny from **a**-α bisexual reproduction between strains 431α and XL280**a** (Table 2).

For each marker, the PCR reaction was carried out using Promega 2× Go Taq Master Mix according to the manufacturer's instructions, and with the following PCR thermal cycles: first an initial denaturation at 94°C for 6 minutes; then 36 cycles of 45 seconds at 94°C, 45 seconds at 60°C, and 90 seconds at 72°C; and a final extension at 72°C for 7 minutes. All enzyme digestions were performed using enzymes purchased from New England Biolabs and following the manufacturer's instructions.

### Genome sequencing, genome assembly, and ploidy determination

Whole genome sequencing was performed using the Illumina platform with Hiseq2500 at the University of North Carolina – Chapel Hill Next Generation Sequencing Facility. A paired end library with approximately 300 base inserts was constructed for each sample, and all libraries were multiplexed and run in one lane using a read length of 100 bases from either side. The Illumina Pipeline (v.1.8.2) was used for initial processing of sequencing data. Sequences have been deposited to the Sequence Read Archive (SRA) under project accession number: SRP042617.

To assemble the genome from each sample, the Illumina reads were first mapped to the JEC21 reference genome [41] • using the short read component of the BWA aligner [42] •. Further refinement was performed using the Genome Analysis Toolkit (GATK) pipeline version 2.4-9 [43] •, including SAMtools [44] • and Picard. Depth of coverage was determined using Qualimap v.0.8 [45].

SNP calling was performed using the GATK Unified Genotyper [43], with the ploidy set to 2, in order to allow calling of both homozygous and heterozygous SNPs. SNPs were visualized using the Broad Integrative Genome Viewer [46].

### Genetic linkage map construction

Linkage maps were constructed using the software MSTmap according to the developers' instructions [47]. For the linkage map of α-α unisexual reproduction, 42 genetic makers (Table 2) and all of the meiotic progeny that were not apparently disomic for chromosome 4 (N = 144; S5 Table) were utilized. For **a**-α bisexual reproduction, a total of 18 genetic markers (17 loci; Table 2), as well as one progeny for each unique genotype identified among the meiotic products of **a**-α bisexual reproduction (N = 76; Table 3), were included for the linkage map construction.

### CHEF gel electrophoresis

DNA plugs for CHEF gel electrophoresis were prepared as previously described [40]. The CHEF gels were run using 1% agarose gel in 0.5×TBE at 14°C for 96 hours, with a ramped switching time from 260 seconds to 560 seconds, at a voltage of 3 V/cm.

### FACS analysis

The ploidy of the parental strains, as well as selected meiotic progeny from α-α unisexual and **a**-α bisexual reproduction, was determined by flow cytometry following a protocol reported previously [48].

## Supporting Information

S1 Figure Ploidy of the meiotic progeny disomic for chromosome 4 were close to diploid by FACS analyses. The three panels in the top row are FACS analyses of the three parental strains: 431α, XL280αSS, and XL280**a**. The panels in rows 2 to 5 are FACS analyses of the meiotic progeny from α-α unisexual and **a**-α bisexual reproduction that were disomic for chromosome 4 (see Fig. 6). The two panels within each dashed-lined rectangle are FACS analyses of the small and large colonies derived from the same meiotic products.
(TIFF)

S2 Figure Meiotic progeny disomic for chromosome 4 are diploid or aneuploid based on genome sequencing. For each panel, the numbers within the columns separated by dashed lines indicated chromosome numbers in the reference genome JEC21. The numbers on the Y-axis indicate the read depth from the whole genome sequencing. The three panels in S2a Figure are the three parental strains: 431α, XL280αSS, and XL280**a**. The panels in S2b–S2k Figure are the meiotic progeny from α-α unisexual and **a**-α bisexual reproduction that were disomic for chromosome 4 (see Fig. 6). For S2b, c, e, f, h Figure the two panels within each Figure are the small and large colonies derived from the same meiotic progeny.
(TIF)

S3 Figure The SNP distribution in meiotic progeny disomic for chromosome 4. The SNPs were identified by comparing the genome sequences of the progeny, as well as their parental strains, against the published genome sequence of strain JEC21. The light blue bars indicate SNPs compared to JEC21, the dark blue bars indicated sites that are heterozygous, and the grey areas indicate regions that are identical to JEC21. S3a Figure shows chromosomes 1 to 4, S3b Figure shows chromosomes 5 to 8, S3c Figure shows chromosomes 9 to 12, and S3d Figure shows chromosomes 13 and 14.
(TIF)

S4 Figure Serotype and mating type verification of the strains studied. A) Strains 431α and XL280αSS have similar karyotypic profiles. B) Serotype D, mating type **a** specific primers fail to amplify from either *MAT*α strain 431α or XL280αSS. C) Serotype D, mating type α specific primers amplify from both strains 431α and XL280αSS, although No. 9 and No. 12 produced PCR products that are smaller from strain 431α than those from strain XL280αSS. For B) and C), the sample order for each PCR was (from left to right): 1 kb DNA ladder, 431α, XL280αSS, XL280**a**, JEC20**a**, JEC21α, and 1 kb DNA ladder, in which JEC20**a** and JEC21α served as positive controls for *MAT* **a** and *MAT*α alleles, respectively.
(TIFF)

S1 Table Phenotypic segregation among progeny from α-α unisexual reproduction.
(DOCX)

S2 Table Phenotypic segregation among progeny from **a**-α bisexual reproduction.
(DOCX)

S3 Table Genotypes of the progeny from α-α unisexual reproduction between strains 431α and XL280αSS.
(DOCX)

S4 Table Genotypes of the progeny from **a**-α bisexual reproduction between strains 431α and XL280**a**.
(DOCX)

S5 Table Distribution of the numbers of detected crossovers along chromosome 4.
(DOCX)

S6 Table Serotype D mating type specific PCR primers employed for strain confirmation.
(DOCX)
