# Supplementary figures and images for: Unisexual Reproduction Drives Meiotic Recombination and Phenotypic and Karyotypic Plasticity in Cryptococcus neoformans
Source: PLoS Genet. 2014 Dec 11;10(12):e1004849. doi: 10.1371/journal.pgen.1004849 (PMC4263396; doi:10.1371/journal.pgen.1004849)

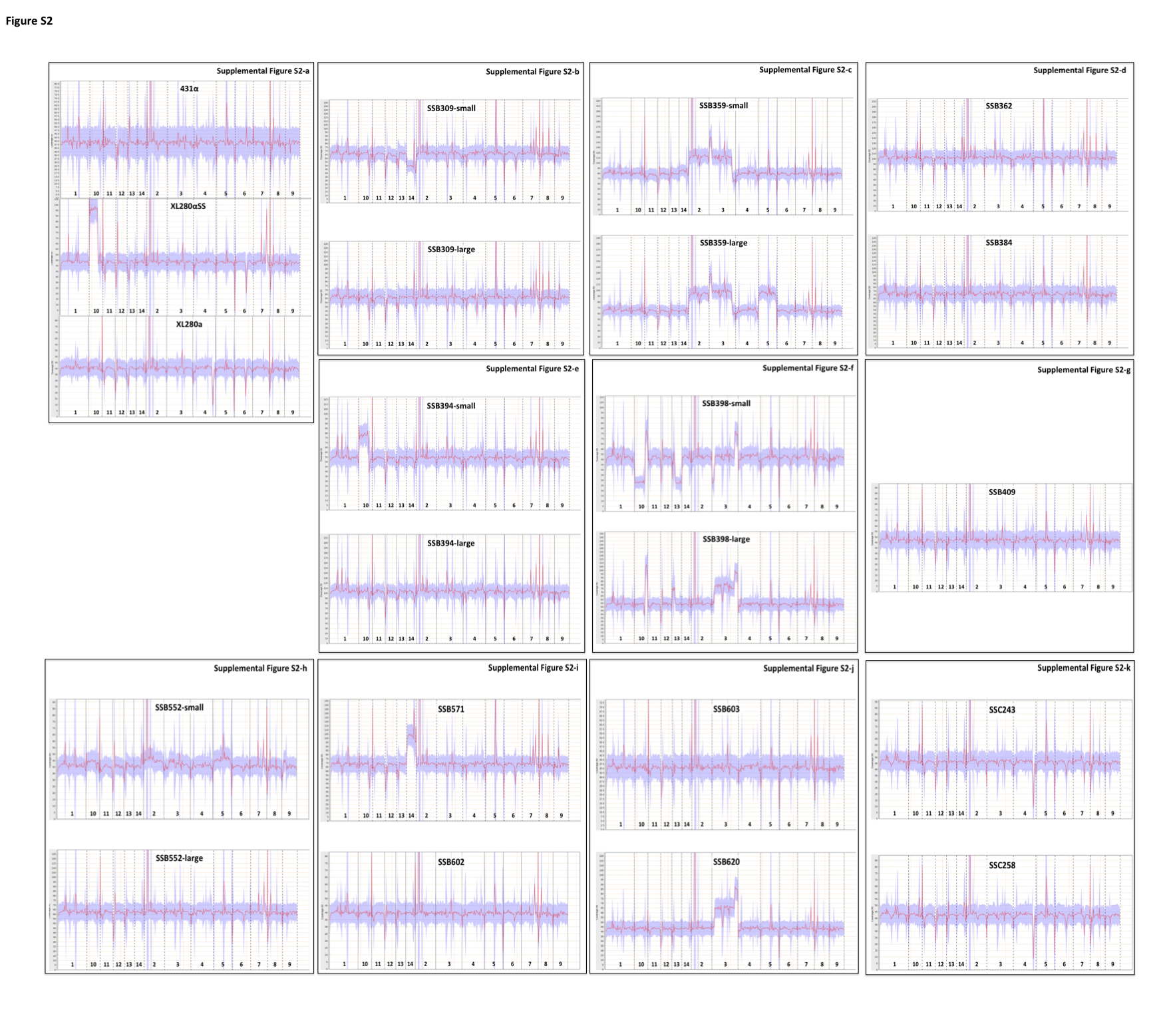

Supplement: S2 Figure — Meiotic progeny disomic for chromosome 4 are diploid or aneuploid based on genome sequencing. For each panel, the numbers within the columns separated by dashed lines indicated chromosome numbers in the reference genome JEC21. The numbers on the Y-axis indicate the read depth from the whole genome sequencing. The three panels in S2a Figure are the three parental strains: 431α, XL280αSS, and XL280a. The panels in S2b–S2k Figure are the meiotic progeny from α-α unisexual and a-α bisexual reproduction that were disomic for chromosome 4 (see Fig. 6). For S2b, c, e, f, h Figure the two panels within each Figure are the small and large colonies derived from the same meiotic progeny. (TIF) [file pgen.1004849.s002.tif]

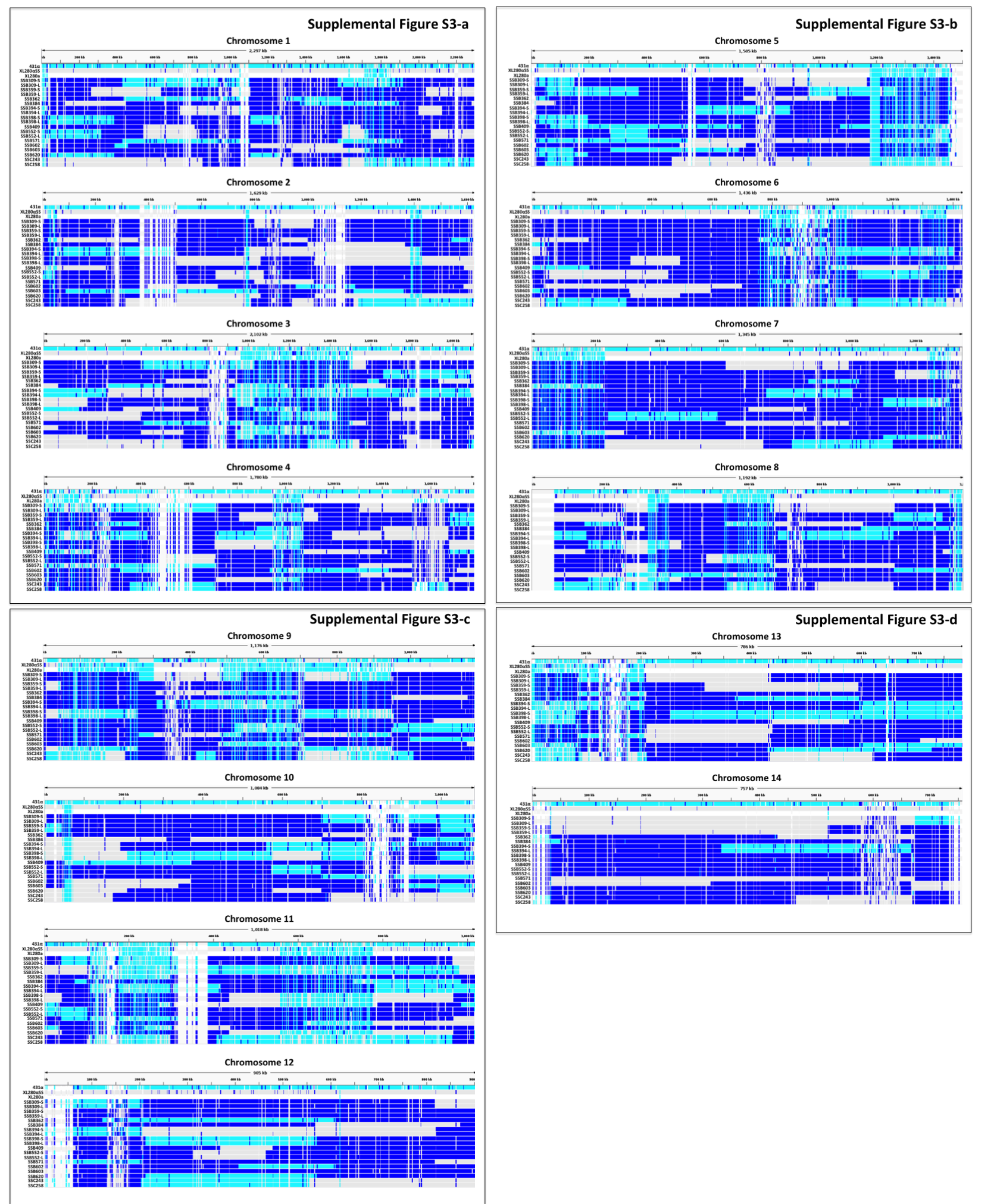

Supplement: S3 Figure — The SNP distribution in meiotic progeny disomic for chromosome 4. The SNPs were identified by comparing the genome sequences of the progeny, as well as their parental strains, against the published genome sequence of strain JEC21. The light blue bars indicate SNPs compared to JEC21, the dark blue bars indicated sites that are heterozygous, and the grey areas indicate regions that are identical to JEC21. S3a Figure shows chromosomes 1 to 4, S3b Figure shows chromosomes 5 to 8, S3c Figure shows chromosomes 9 to 12, and S3d Figure shows chromosomes 13 and 14. (TIF) [file pgen.1004849.s003.tif]

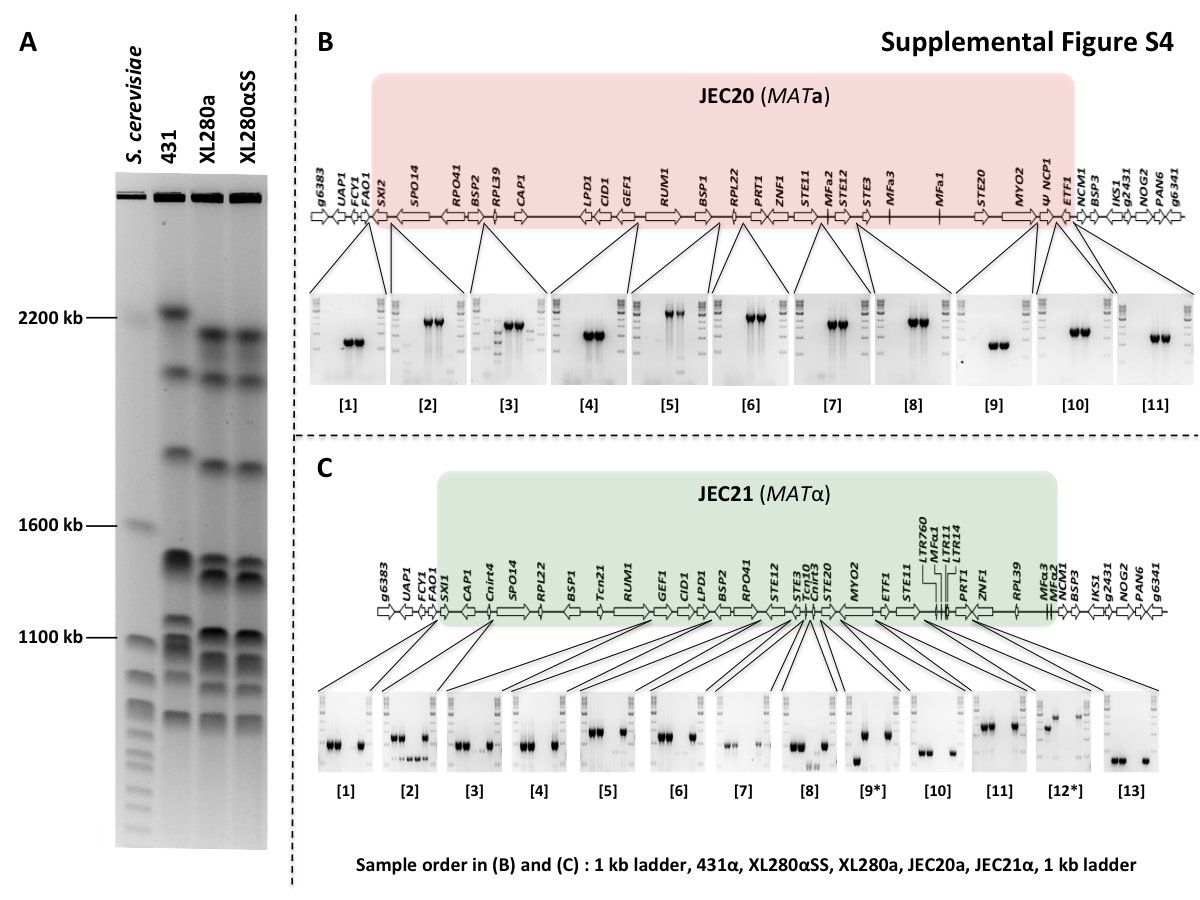

Supplement: S4 Figure — Serotype and mating type verification of the strains studied. A) Strains 431α and XL280αSS have similar karyotypic profiles. B) Serotype D, mating type a specific primers fail to amplify from either MATα strain 431α or XL280αSS. C) Serotype D, mating type α specific primers amplify from both strains 431α and XL280αSS, although No. 9 and No. 12 produced PCR products that are smaller from strain 431α than those from strain XL280αSS. For B) and C), the sample order for each PCR was (from left to right): 1 kb DNA ladder, 431α, XL280αSS, XL280a, JEC20a, JEC21α, and 1 kb DNA ladder, in which JEC20a and JEC21α served as positive controls for MAT a and MATα alleles, respectively. (TIFF) [file pgen.1004849.s004.tiff]
