# Supplementary material for: Unisexual Reproduction Drives Meiotic Recombination and Phenotypic and Karyotypic Plasticity in Cryptococcus neoformans
Source: PLoS Genet. 2014 Dec 11;10(12):e1004849. doi: 10.1371/journal.pgen.1004849 (PMC4263396; doi:10.1371/journal.pgen.1004849)
Supplement: S1 Table — Phenotypic segregation among progeny from α-α unisexual reproduction. (DOCX) [file pgen.1004849.s005.docx]

**Table S1. Phenotypic segregation among progeny from α-α unisexual reproduction**

| **Strain** | **YPD_30°C** | **YPD_37°C** | **YPD_40°C** | **YPD_41°C** | **L-DOPA** | **MS** |
| --- | --- | --- | --- | --- | --- | --- |
| 431α | +++ | +++ | + | - | + | - |
| XL280αSS | +++ | +++ | - | - | - | ++ |
| SSB307 | +++ | +++ | - | - | - | - |
| SSB308 | +++ | +++ | + | + | - | - |
| SSB309 | +++ | +++ | ++ | + | - | - |
| SSB310 | +++ | + | - | - | +++ | - |
| SSB311 | +++ | +++ | +++ | + | ++ | - |
| SSB312 | +++ | +++ | + | + | - | - |
| SSB313 | +++ | +++ | +++ | + | - | - |
| SSB314 | +++ | ++ | + | + | +++ | - |
| SSB315 | +++ | +++ | + | + | - | - |
| SSB316 | +++ | +++ | + | - | +++ | - |
| SSB317 | +++ | +++ | +++ | + | - | - |
| SSB349 | +++ | +++ | - | - | + | - |
| SSB350 | +++ | +++ | - | - | + | - |
| SSB351 | +++ | +++ | - | - | + | - |
| SSB352 | +++ | +++ | - | - | + | - |
| SSB353 | +++ | +++ | - | - | + | - |
| SSB354 | +++ | +++ | +++ | - | - | - |
| SSB355 | +++ | +++ | +++ | - | ++ | - |
| SSB356 | +++ | +++ | +++ | + | - | - |
| SSB357 | +++ | +++ | - | - | - | + |
| SSB358 | +++ | +++ | - | - | + | - |
| SSB359 | +++ | +++ | +++ | - | - | - |
| SSB360 | +++ | +++ | + | - | ++ | - |
| SSB361 | +++ | +++ | +++ | - | - | - |
| SSB362 | +++ | +++ | + | - | +++ | - |
| SSB363 | +++ | +++ | ++ | - | ++ | - |
| SSB364 | +++ | +++ | ++ | - | ++ | - |
| SSB365 | +++ | +++ | + | - | - | - |
| SSB366 | +++ | +++ | - | - | - | + |
| SSB367 | +++ | +++ | ++ | - | ++ | - |
| SSB368 | +++ | +++ | - | - | + | - |
| SSB369 | +++ | +++ | +++ | + | - | - |
| SSB370 | +++ | +++ | +++ | - | - | - |
| SSB371 | +++ | +++ | +++ | - | - | - |
| SSB372 | +++ | +++ | + | + | - | - |
| SSB373 | +++ | +++ | +++ | + | - | - |
| SSB374 | +++ | +++ | ++ | - | +++ | - |
| SSB375 | +++ | +++ | +++ | + | - | - |
| SSB376 | +++ | +++ | +++ | + | - | + |
| SSB377 | +++ | +++ | + | + | - | - |
| SSB378 | +++ | +++ | +++ | + | - | - |
| SSB379 | +++ | +++ | + | - | - | - |
| SSB380 | +++ | +++ | +++ | - | - | - |
| SSB381 | +++ | +++ | + | - | + | - |
| SSB382 | +++ | +++ | - | - | +++ | - |
| SSB383 | +++ | +++ | ++ | - | + | + |
| SSB384 | +++ | +++ | +++ | + | ++ | - |
| SSB385 | +++ | +++ | +++ | + | - | - |
| SSB386 | +++ | +++ | +++ | + | - | - |
| SSB387 | +++ | + | - | - | - | + |
| SSB388 | +++ | +++ | + | - | - | - |
| SSB389 | +++ | +++ | ++ | - | + | + |
| SSB390 | +++ | +++ | + | + | - | - |
| SSB391 | +++ | +++ | ++ | + | + | - |
| SSB392 | +++ | +++ | - | - | - | - |
| SSB393 | +++ | +++ | - | - | - | + |
| SSB394 | +++ | +++ | ++ | - | ++ | - |
| SSB395 | +++ | +++ | +++ | + | - | - |
| SSB396 | +++ | +++ | ++ | - | - | - |
| SSB397 | +++ | +++ | ++ | - | - | - |
| SSB398 | +++ | +++ | ++ | - | + | - |
| SSB399 | +++ | +++ | - | - | - | - |
| SSB400 | +++ | +++ | - | - | - | + |
| SSB401 | +++ | +++ | ++ | - | - | - |
| SSB402 | +++ | +++ | ++ | + | - | - |
| SSB403 | +++ | +++ | +++ | + | - | - |
| SSB404 | +++ | +++ | ++ | + | - | - |
| SSB405 | +++ | +++ | ++ | - | - | - |
| SSB406 | +++ | +++ | + | + | - | - |
| SSB407 | +++ | +++ | + | + | ++ | ++ |
| SSB408 | +++ | +++ | + | + | - | - |
| SSB409 | +++ | +++ | +++ | + | + | - |
| SSB410 | +++ | - | - | - | +++ | - |
| SSB411 | +++ | +++ | +++ | + | - | - |
| SSB412 | +++ | +++ | ++ | + | ++ | - |
| SSB549 | +++ | +++ | + | + | - | - |
| SSB550 | +++ | +++ | + | + | - | - |
| SSB551 | +++ | +++ | + | - | - | - |
| SSB552 | +++ | +++ | +++ | + | ++ | - |
| SSB553 | +++ | +++ | + | - | ++ | ++ |
| SSB554 | +++ | +++ | +++ | ++ | - | - |
| SSB555 | +++ | +++ | ++ | - | - | - |
| SSB556 | +++ | +++ | +++ | ++ | - | - |
| SSB557 | +++ | +++ | + | - | - | - |
| SSB558 | +++ | +++ | - | - | - | - |
| SSB559 | +++ | +++ | ++ | - | ++ | - |
| SSB560 | +++ | +++ | - | - | - | - |
| SSB561 | +++ | +++ | ++ | - | ++ | - |
| SSB562 | +++ | +++ | ++ | - | - | + |
| SSB563 | +++ | +++ | ++ | - | - | - |
| SSB564 | +++ | ++ | - | - | + | - |
| SSB565 | +++ | +++ | +++ | + | - | - |
| SSB566 | +++ | +++ | ++ | - | - | - |
| SSB567 | +++ | +++ | ++ | - | - | - |
| SSB568 | +++ | +++ | ++ | - | - | - |
| SSB569 | +++ | +++ | ++ | - | - | - |
| SSB570 | +++ | +++ | ++ | + | ++ | + |
| SSB571 | +++ | +++ | ++ | + | - | - |
| SSB572 | +++ | +++ | +++ | + | +++ | - |
| SSB573 | +++ | +++ | - | - | - | + |
| SSB574 | +++ | + | - | - | +++ | - |
| SSB575 | +++ | +++ | ++ | - | - | - |
| SSB576 | +++ | +++ | ++ | - | - | - |
| SSB577 | +++ | +++ | ++ | - | - | - |
| SSB578 | +++ | +++ | + | - | - | - |
| SSB579 | +++ | +++ | +++ | + | - | - |
| SSB580 | +++ | +++ | - | - | + | + |
| SSB581 | +++ | +++ | - | - | + | - |
| SSB582 | +++ | +++ | +++ | ++ | - | - |
| SSB583 | +++ | +++ | - | - | + | - |
| SSB584 | +++ | +++ | - | - | + | - |
| SSB585 | +++ | +++ | - | - | +++ | ++ |
| SSB586 | +++ | +++ | + | - | + | - |
| SSB587 | +++ | +++ | + | - | ++ | ++ |
| SSB588 | +++ | +++ | - | - | - | - |
| SSB589 | +++ | +++ | +++ | ++ | - | - |
| SSB590 | +++ | +++ | ++ | - | - | - |
| SSB591 | +++ | +++ | ++ | - | - | - |
| SSB592 | +++ | +++ | - | - | + | - |
| SSB593 | +++ | +++ | - | - | ++ | ++ |
| SSB594 | +++ | +++ | ++ | + | - | - |
| SSB595 | +++ | +++ | + | - | - | - |
| SSB596 | +++ | +++ | ++ | - | +++ | - |
| SSB597 | +++ | +++ | + | - | +++ | - |
| SSB598 | +++ | +++ | ++ | - | ++ | ++ |
| SSB599 | +++ | +++ | ++ | - | - | - |
| SSB600 | +++ | +++ | +++ | + | + | - |
| SSB601 | +++ | +++ | ++ | - | - | + |
| SSB602 | +++ | +++ | +++ | ++ | + | - |
| SSB603 | +++ | +++ | ++ | - | + | - |
| SSB604 | +++ | +++ | + | - | - | - |
| SSB605 | +++ | +++ | - | - | + | - |
| SSB606 | +++ | +++ | - | - | - | - |
| SSB607 | +++ | +++ | +++ | + | + | - |
| SSB608 | +++ | +++ | ++ | - | - | - |
| SSB609 | +++ | + | - | - | +++ | - |
| SSB610 | +++ | +++ | - | - | + | - |
| SSB611 | +++ | +++ | ++ | + | - | - |
| SSB612 | +++ | +++ | ++ | - | - | + |
| SSB613 | +++ | +++ | - | - | + | - |
| SSB614 | +++ | +++ | ++ | - | ++ | - |
| SSB615 | +++ | ++ | + | - | + | - |
| SSB616 | +++ | ++ | - | - | - | + |
| SSB617 | +++ | +++ | ++ | - | - | + |
| SSB618 | +++ | ++ | + | - | + | - |
| SSB619 | +++ | +++ | +++ | + | - | - |
| SSB620 | +++ | +++ | +++ | + | ++ | - |
| SSB621 | +++ | +++ | - | - | +++ | - |
| SSB622 | +++ | +++ | +++ | ++ | - | - |
| SSB623 | +++ | +++ | - | - | - | - |
| SSB624 | +++ | +++ | +++ | + | - | - |
| SSB625 | +++ | +++ | +++ | + | - | - |
| SSB626 | +++ | +++ | +++ | + | +++ | - |
| SSB627 | +++ | +++ | ++ | - | - | - |
| SSB628 | +++ | +++ | +++ | + | - | - |
| SSB629 | +++ | +++ | + | - | + | - |
| SSB630 | +++ | +++ | ++ | - | + | - |
| SSB631 | +++ | +++ | - | - | - | + |
| SSB632 | +++ | +++ | - | - | - | - |
| SSB633 | +++ | +++ | + | - | ++ | ++ |
| SSB634 | +++ | ++ | - | - | + | - |
| SSB635 | +++ | +++ | - | - | + | - |
| SSB636 | +++ | ++ | - | - | +++ | - |
| SSB637 | +++ | +++ | +++ | ++ | - | - |
| SSB638 | +++ | +++ | + | - | + | - |
| SSB639 | +++ | +++ | ++ | + | ++ | - |
| SSB640 | +++ | +++ | +++ | + | ++ | - |
| SSB641 | +++ | +++ | ++ | + | ++ | - |
| SSB642 | +++ | +++ | + | - | - | ++ |
| SSB643 | +++ | +++ | + | + | - | + |
| SSB644 | +++ | +++ | + | + | - | - |

Notes: “-”, “+”, “++”, and “+++” represent different phenotypes as illustrated in Figure 1.
