# Supplementary material for: Unisexual Reproduction Drives Meiotic Recombination and Phenotypic and Karyotypic Plasticity in Cryptococcus neoformans
Source: PLoS Genet. 2014 Dec 11;10(12):e1004849. doi: 10.1371/journal.pgen.1004849 (PMC4263396; doi:10.1371/journal.pgen.1004849)
Supplement: S2 Table — Phenotypic segregation among progeny from a-α bisexual reproduction. (DOCX) [file pgen.1004849.s006.docx]

**Table S2. Phenotypic segregation among progeny from a-α bisexual reproduction**

| **Strain** | **YPD_30°C** | **YPD_37°C** | **YPD_40°C** | **YPD_41°C** | **L-DOPA** | **MS** |
| --- | --- | --- | --- | --- | --- | --- |
| 431α | +++ | +++ | + | - | + | - |
| XL280**a** | +++ | +++ | - | - | - | ++ |
| SSB862 | +++ | +++ | - | - | +++ | - |
| SSB864 | +++ | +++ | + | + | - | - |
| SSB868 | +++ | + | - | - | + | - |
| SSB869 | +++ | + | - | - | + | - |
| SSB872 | +++ | + | - | - | + | - |
| SSB873 | +++ | + | - | - | + | - |
| SSB874 | +++ | +++ | + | - | - | ++ |
| SSB876 | +++ | +++ | ++ | - | - | ++ |
| SSB879 | +++ | +++ | - | - | + | - |
| SSB880 | +++ | +++ | +++ | + | - | ++ |
| SSB885 | +++ | +++ | - | - | + | - |
| SSB886 | +++ | +++ | +++ | + | - | ++ |
| SSB887 | +++ | +++ | - | - | + | - |
| SSB889 | +++ | ++ | - | - | + | - |
| SSB890 | +++ | +++ | - | - | - | ++ |
| SSB892 | +++ | +++ | - | - | - | ++ |
| SSB896 | +++ | +++ | - | - | - | ++ |
| SSB898 | +++ | +++ | - | - | - | - |
| SSB901 | +++ | +++ | - | - | - | ++ |
| SSB904 | +++ | + | - | - | + | - |
| SSB905 | +++ | ++ | - | - | + | - |
| SSB906 | +++ | ++ | - | - | + | - |
| SSB908 | +++ | +++ | ++ | - | - | ++ |
| SSB926 | +++ | ++ | + | - | + | - |
| SSB927 | +++ | +++ | + | - | ++ | - |
| SSB929 | +++ | +++ | +++ | + | + | ++ |
| SSB930 | +++ | +++ | - | - | + | - |
| SSB952 | +++ | +++ | +++ | - | + | - |
| SSB956 | +++ | +++ | +++ | - | - | ++ |
| SSB960 | +++ | +++ | +++ | - | - | ++ |
| SSB961 | +++ | +++ | +++ | - | - | ++ |
| SSB963 | +++ | +++ | + | - | + | - |
| SSB966 | +++ | +++ | + | - | ++ | - |
| SSB969 | +++ | +++ | - | - | + | - |
| SSB972 | +++ | +++ | + | - | - | + |
| SSB976 | +++ | - | - | - | +++ | - |
| SSB977 | +++ | - | - | - | +++ | - |
| SSB980 | +++ | +++ | + | - | - | + |
| SSB984 | +++ | +++ | + | - | - | + |
| SSB996 | +++ | +++ | - | - | +++ | - |
| SSB997 | +++ | +++ | - | - | ++ | - |
| SSB998 | +++ | +++ | - | - | + | - |
| SSB999 | +++ | +++ | - | - | ++ | - |
| SSC001 | +++ | +++ | +++ | - | +++ | - |
| SSC007 | +++ | ++ | - | - | + | - |
| SSC008 | +++ | ++ | - | - | + | - |
| SSC017 | +++ | +++ | - | - | + | - |
| SSC023 | +++ | +++ | + | - | +++ | - |
| SSC026 | +++ | ++ | - | - | + | - |
| SSC029 | +++ | ++ | - | - | + | - |
| SSC030 | +++ | +++ | - | - | +++ | - |
| SSC031 | +++ | +++ | - | - | +++ | - |
| SSC033 | +++ | +++ | - | - | +++ | - |
| SSC036 | +++ | +++ | - | - | + | - |
| SSC039 | +++ | +++ | + | - | + | - |
| SSC040 | +++ | +++ | ++ | - | + | - |
| SSC045 | +++ | +++ | ++ | - | + | - |
| SSC047 | +++ | +++ | ++ | - | + | - |
| SSC049 | +++ | +++ | ++ | - | + | - |
| SSC050 | +++ | +++ | - | - | - | ++ |
| SSC228 | +++ | +++ | +++ | - | + | ++ |
| SSC235 | +++ | +++ | - | - | ++ | + |
| SSC237 | +++ | +++ | - | - | + | + |
| SSC239 | +++ | +++ | +++ | - | ++ | - |
| SSC242 | +++ | +++ | +++ | + | + | ++ |
| SSC243 | +++ | +++ | +++ | - | + | - |
| SSC245 | +++ | +++ | +++ | + | - | - |
| SSC248 | +++ | ++ | - | - | + | - |
| SSC249 | +++ | +++ | ++ | - | ++ | - |
| SSC252 | +++ | +++ | +++ | + | - | - |
| SSC258 | +++ | +++ | ++ | - | + | - |
| SSC260 | +++ | +++ | +++ | - | + | ++ |
| SSC271 | +++ | +++ | +++ | - | + | ++ |
| SSC272 | +++ | +++ | ++ | - | ++ | - |
| SSC273 | +++ | +++ | - | - | + | - |
| SSC278 | +++ | ++ | - | - | +++ | - |
| SSC279 | +++ | ++ | - | + | ++ | ++ |
| SSC281 | +++ | +++ | - | - | + | - |
| SSC286 | +++ | +++ | - | - | - | ++ |
| SSC290 | +++ | +++ | + | - | ++ | - |
| SSC291 | +++ | +++ | + | - | + | ++ |

Notes: “-”, “+”, “++”, and “+++” represent different phenotypes as illustrated in Figure 1.
