# Supplementary material for: Unisexual Reproduction Drives Meiotic Recombination and Phenotypic and Karyotypic Plasticity in Cryptococcus neoformans
Source: PLoS Genet. 2014 Dec 11;10(12):e1004849. doi: 10.1371/journal.pgen.1004849 (PMC4263396; doi:10.1371/journal.pgen.1004849)
Supplement: S3 Table — Genotypes of the progeny from α-α unisexual reproduction between strains 431α and XL280αSS. (DOCX) [file pgen.1004849.s007.docx]

**Table S3. Genotypes of the progeny from α-α unisexual reproduction between strains 431α and XL280αSS.**


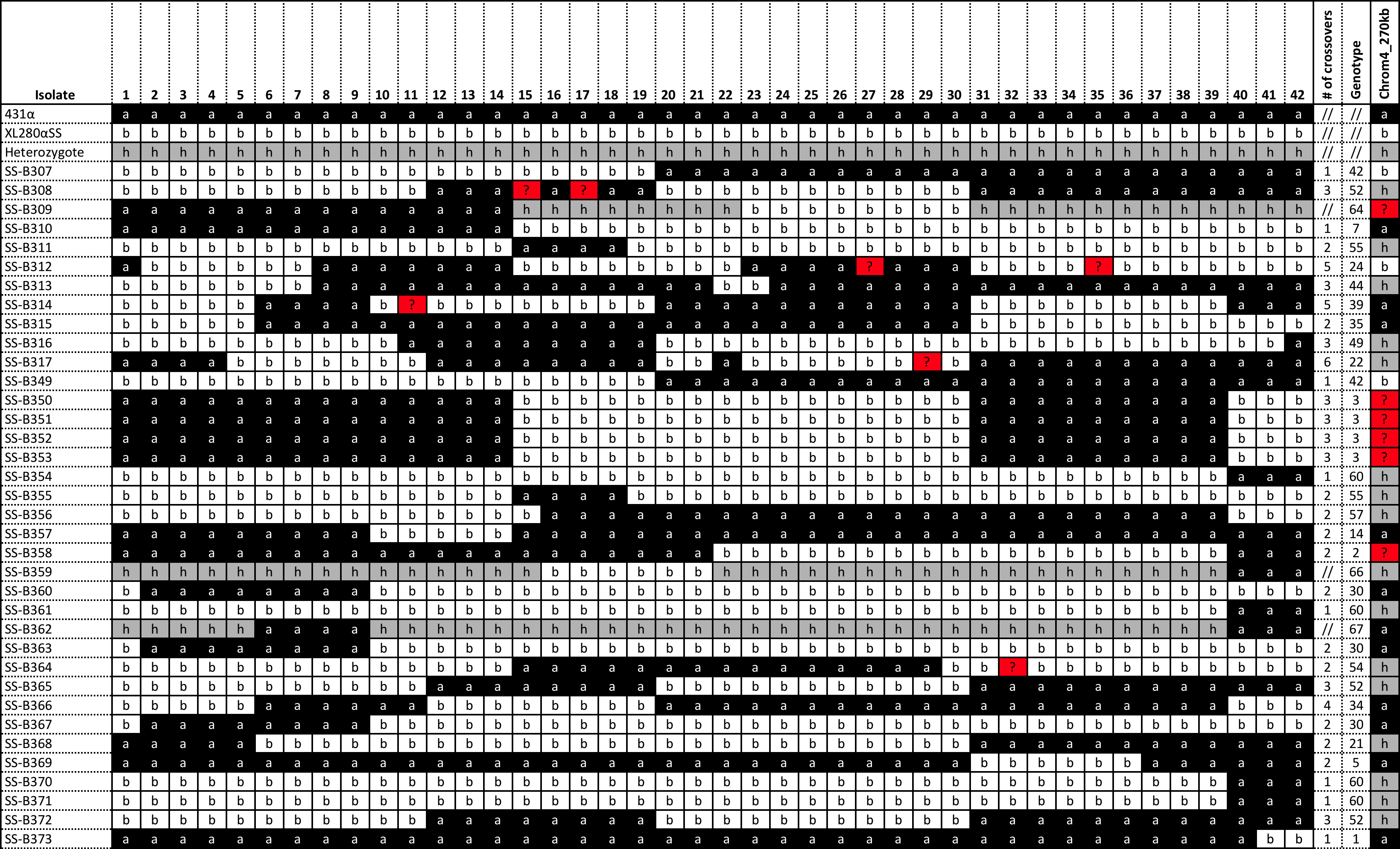


***(Table S3. Continued)***


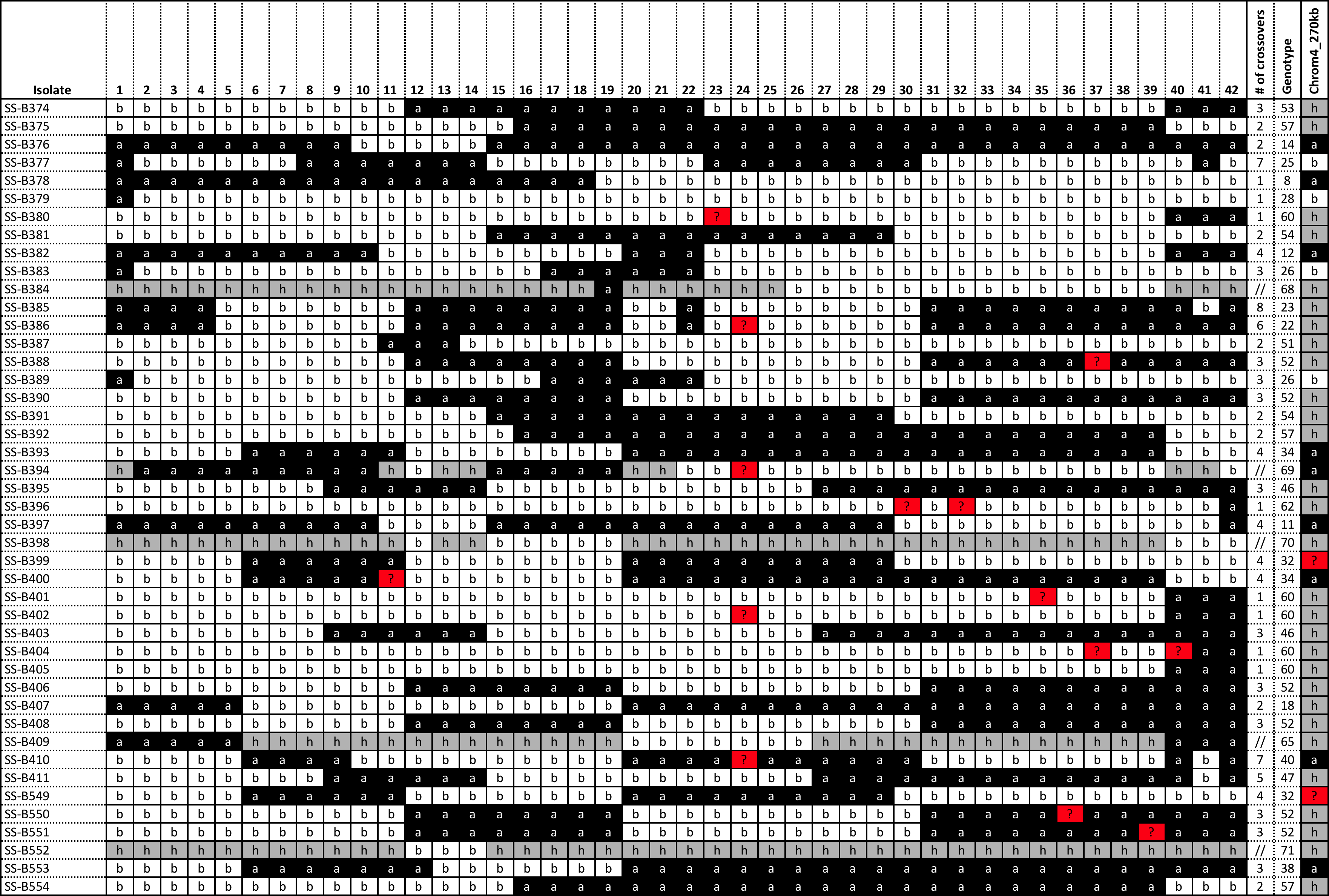


***(Table S3. Continued)***


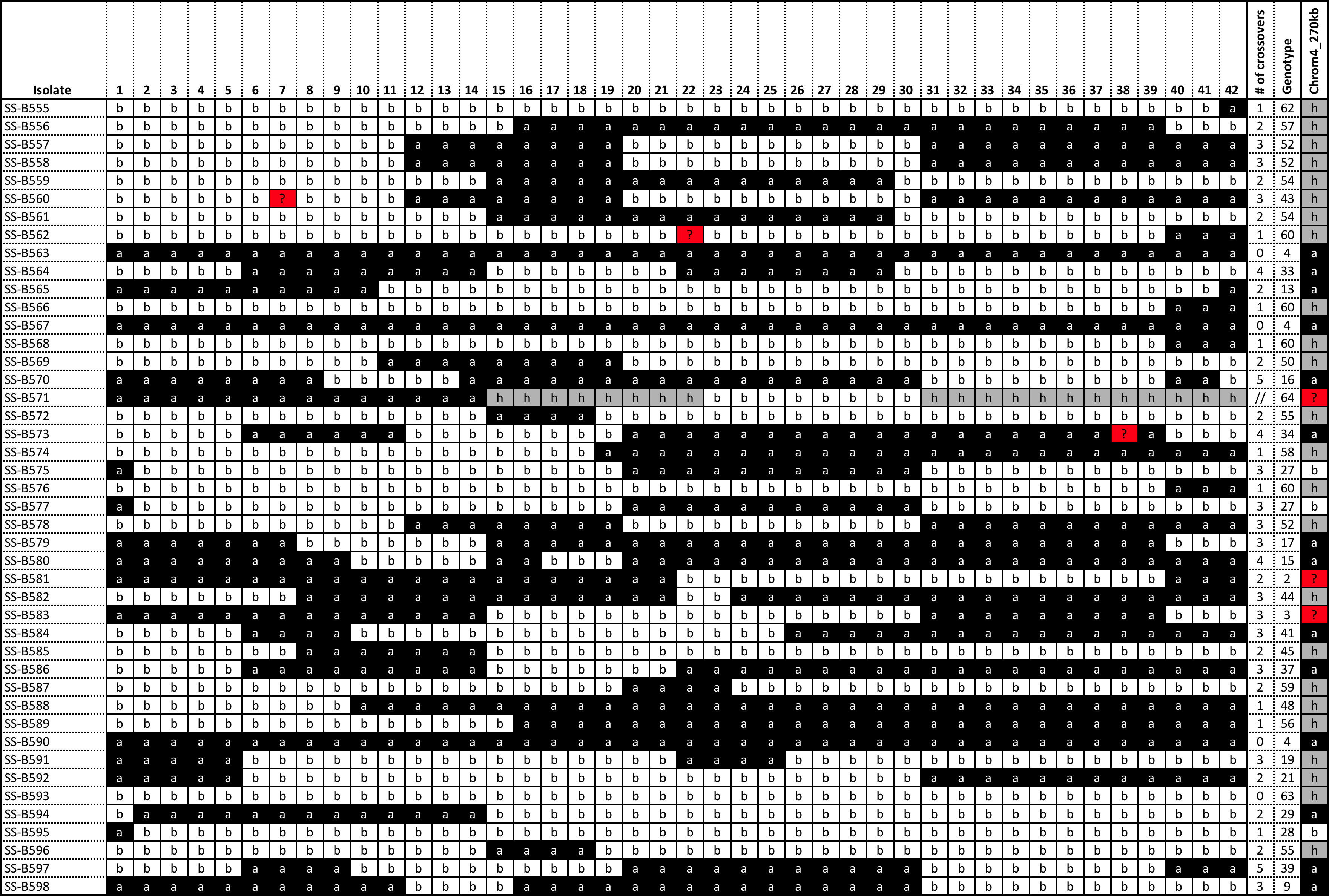


***(Table S3. Continued)***


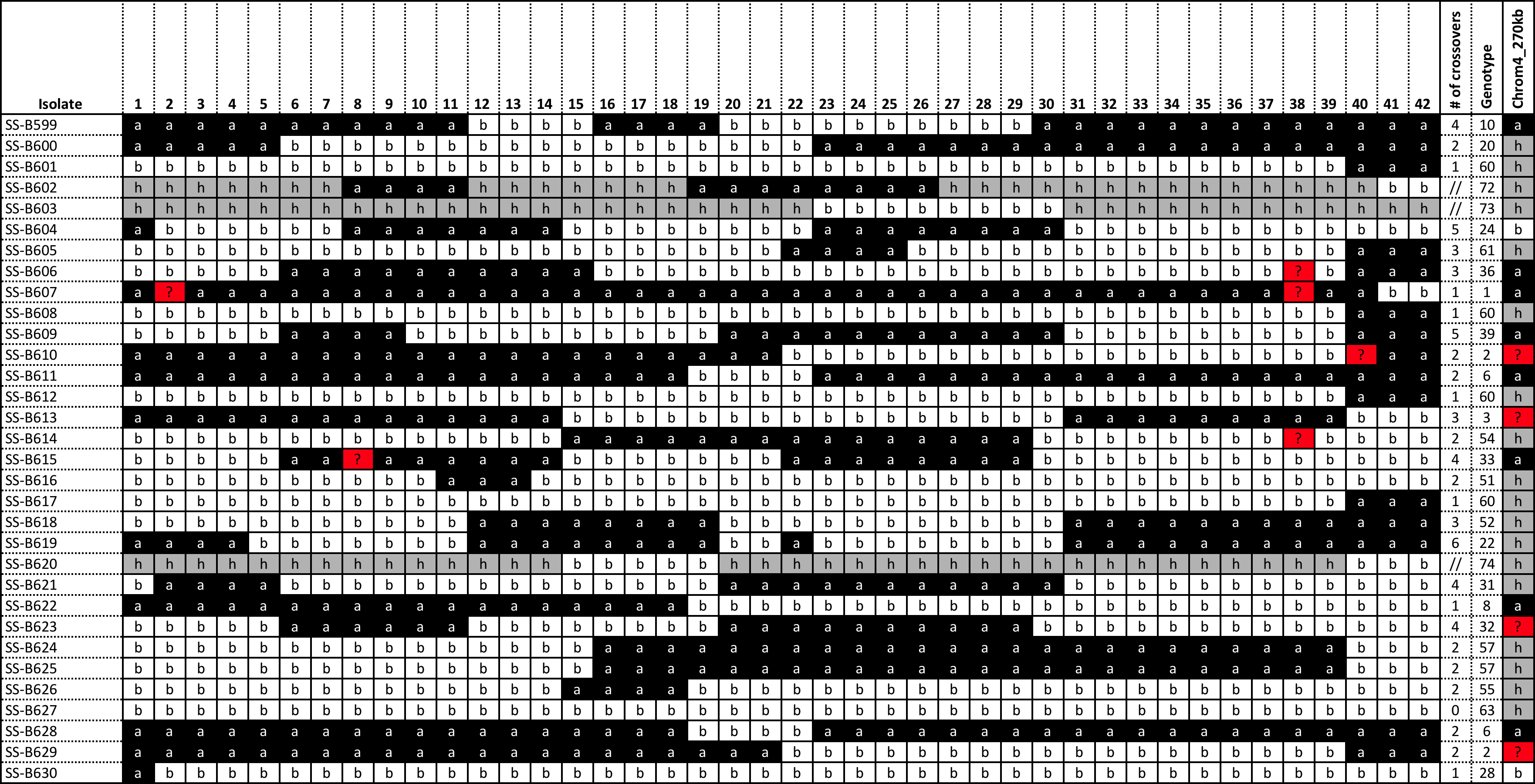


Notes: “a” indicates the allele from the parental strain 431α; “b” indicates the allele from the parental strain XL280αSS; “h” indicates heterozygous with both alleles; and “?” indicates missing data.
