# Supplementary material for: Unisexual Reproduction Drives Meiotic Recombination and Phenotypic and Karyotypic Plasticity in Cryptococcus neoformans
Source: PLoS Genet. 2014 Dec 11;10(12):e1004849. doi: 10.1371/journal.pgen.1004849 (PMC4263396; doi:10.1371/journal.pgen.1004849)
Supplement: S4 Table — Genotypes of the progeny from a-α bisexual reproduction between strains 431α and XL280a. (DOCX) [file pgen.1004849.s008.docx]

**Table S4. Genotypes of the progeny from a-α bisexual reproduction between strains 431α and XL280a.**

***(Table S4. Continued)***

Notes: “a” indicates the allele from the parental strain 431α; “b” indicates the allele from the parental strain XL280**a**; “h” indicates heterozygous with both alleles; and “?” indicates missing data.
