# Supplementary material for: Unisexual Reproduction Drives Meiotic Recombination and Phenotypic and Karyotypic Plasticity in Cryptococcus neoformans
Source: PLoS Genet. 2014 Dec 11;10(12):e1004849. doi: 10.1371/journal.pgen.1004849 (PMC4263396; doi:10.1371/journal.pgen.1004849)
Supplement: S5 Table — Distribution of the numbers of detected crossovers along chromosome 4. (DOCX) [file pgen.1004849.s009.docx]

**Table S5. Distribution of the numbers of detected crossovers along chromosome 4**

| **No. of crossovers along chromosome 4** | **No. of progeny**  **(α-α unisexual reproduction)** | **No. of progeny**  **(a-α bisexual reproduction)** |
| --- | --- | --- |
| 0 | 5 | 4 |
| 1 | 32 | 14 |
| 2 | 42 | 21 |
| 3 | 38 | 18 |
| 4 | 14 | 13 |
| 5 | 7 | 4 |
| 6 | 3 | 3 |
| 7 | 2 | 0 |
| 8 | 1 | 0 |
