# Supplementary material for: Unisexual Reproduction Drives Meiotic Recombination and Phenotypic and Karyotypic Plasticity in Cryptococcus neoformans
Source: PLoS Genet. 2014 Dec 11;10(12):e1004849. doi: 10.1371/journal.pgen.1004849 (PMC4263396; doi:10.1371/journal.pgen.1004849)
Supplement: S6 Table — Serotype D mating type specific PCR primers employed for strain confirmation. (DOCX) [file pgen.1004849.s010.docx]

**Table S6. Serotype D mating type specific PCR primers employed for strain confirmation.**

| Intergenic Region | Primer (Forward) | Primer (Reverse) | Note ^1^ |
| --- | --- | --- | --- |
| *M****A****T***a***_FAO1_SXI2***a** | JOHE40807 | JOHE40808 | B1 |
| *MAT***a***_SXI2***a***_SPO14* | JOHE40809 | JOHE40810 | B2 |
| *MAT***a***_BSP2_RPL39* | JOHE40815 | JOHE40816 | B3 |
| *MAT***a***_GEF1_RUM1* | JOHE40825 | JOHE40826 | B4 |
| *MAT***a***_BSP1_RPL22* | JOHE40829 | JOHE40830 | B5 |
| *MAT***a***_RPL22_PRT1* | JOHE40831 | JOHE40832 | B6 |
| *MAT***a***_STE11_MF***a**2 | JOHE40837 | JOHE40838 | B7 |
| *MAT***a***_STE12_STE3* | JOHE40841 | JOHE40842 | B8 |
| *MAT***a***_MYO2_NCP1Ψ* | JOHE40851 | JOHE40852 | B9 |
| *MAT***a***_NCP1Ψ_ETF1* | JOHE40853 | JOHE40854 | B10 |
| *MAT***a***_ETF1_NCM1* | JOHE40855 | JOHE40856 | B11 |
|  |  |  |  |
| *MAT*α*_FAO1_SXI1*α | JOHE39896 | JOHE39897 | C1 |
| *MAT*α*_LTR-Cnirt4_SPO14* | JOHE39902 | JOHE39903 | C2 |
| *MAT*α*_RUM1_GEF1* | JOHE39912 | JOHE39913 | C3 |
| *MAT*α*_LPD1_BSP2* | JOHE39918 | JOHE39919 | C4 |
| *MAT*α*_RPO41_STE12* | JOHE39922 | JOHE39923 | C5 |
| *MAT*α*_STE12_STE3* | JOHE39924 | JOHE39925 | C6 |
| *MAT*α*_STE3_LTR-Tcn10* | JOHE39926 | JOHE39927 | C7 |
| *MAT*α*_LTR-Tcn10_LTR-Cnirt3* | JOHE39928 | JOHE39929 | C8 |
| *MAT*α*_LTR-Cnirt3_STE20* | JOHE39930 | JOHE39931 | C9 |
| *MAT*α*_STE20_MYO2* | JOHE39932 | JOHE39933 | C10 |
| *MAT*α*_MYO2_ETF1* | JOHE39934 | JOHE39935 | C11 |
| *MAT*α*_STE11_LTR760* | JOHE39938 | JOHE39939 | C12 |
| *MAT*α*_PRT1_ZNF1* | JOHE39942 | JOHE39943 | C13 |

^1^: B1 – B11 and C1 – C13 correspond to the markers shown in Supplemental Figures S4B and S4C, respectively.
